# Resistance versus concurrent training with three assigned protein targets in middle-aged and older women: a randomized 2 × 3 factorial trial

**DOI:** 10.1080/15502783.2026.2720742

**Published:** 2026-09-14

**Authors:** Reza Bagheri, Hamid Ghobadi, Maryam Mansordehghan, Donny M Camera, Mehdi Kargarfard

**Affiliations:** a Department of Exercise Physiology, University of Isfahan, Isfahan, Iran; b Department of Exercise Physiology, Ferdowsi University of Mashhad, Mashhad, Iran; c Student Research Committee, Nutritional Sciences Department, School of Nutrition Sciences and Food Technology, Kermanshah University of Medical Sciences, Kermanshah, Iran; d Department of Health and Biostatistics, Swinburne University, Melbourne, Australia

**Keywords:** Exercise training, muscular adaptations, nutrition, body composition

## Abstract

**Background:**

Evidence is limited regarding whether assigned protein targets modify responses to resistance training (RT) alone or to the same RT program plus cycling (concurrent training [CT]) in middle-aged and older women. This randomized 2 × 3 factorial trial examined bioelectrical impedance analysis (BIA)-derived skeletal muscle mass (SMM; primary outcome), other body composition outcomes, muscular and functional performance, and cycle-derived estimated VO₂max.

**Methods:**

In this randomized 2 × 3 factorial trial, 108 women aged 40–77 years were assigned to 12 weeks of supervised RT or CT (identical RT followed by cycling) and protein targets of 0.8, 1.6, or 2.2 g·kg^−1^·d^−1^. Baseline-adjusted ANCOVA tested training × protein interactions and marginal training and protein effects. Complete-case analyses included 83 participants.

**Results:**

For SMM, no training-condition × protein-target interaction (*p* = 0.856), marginal protein-target effect (*p* = 0.726), or marginal training-condition effect (*p* = 0.273) was detected. CT had a lower baseline-adjusted week-12 BFP than RT (adjusted difference, −2.04 percentage points; 95% CI, −2.94 to −1.14; *p* < 0.001). RT had a higher baseline-adjusted week-12 leg-press estimated 1-RM than CT (CT − RT: −6.68 kg; 95% CI, −8.32 to −5.04; *p* < 0.001), whereas CT had a higher baseline-adjusted week-12 cycle-derived estimated VO₂max (adjusted difference, 4.53 mL·kg^−1^·min^−1^; 95% CI, 3.80 to 5.25; *p* < 0.001). No detectable marginal protein-target effects or training-condition × protein-target interactions were observed for the key secondary outcomes.

**Conclusions:**

No detectable differences in SMM or key secondary outcomes were attributable to assigned protein target. Compared with RT, CT favored estimated aerobic fitness and BFP, whereas RT favored leg-press strength. Because CT included additional cycling and greater exercise exposure, these differences cannot be attributed solely to training modality. Null protein findings do not establish equivalence among doses.

## 1. Introduction

Population aging is a major global public health challenge, with preservation of physical function, independence, and cardiometabolic health becoming increasingly important across middle and later adulthood [1–3]. Age-related changes in skeletal muscle mass (SMM), body composition, strength, and cardiorespiratory fitness may begin during midlife and progress with advancing age. Women may also experience sex-specific physiological and health challenges across this broad age range, including changes in musculoskeletal and cardiometabolic health [4]. However, because menopausal stage and hormonal status were not characterised in the present study, the population is described according to age rather than presumed menopausal status. These challenges highlight the need for lifestyle strategies that preserve health and independence in women. Rowe and Kahn’s concept of successful aging emphasises low disease risk and strong functioning, later expanded into active aging, which highlights overall well-being [5]. Interventions initiated during midlife may therefore provide an opportunity to preserve physical capacity and attenuate subsequent age-related decline.

Exercise and diet are central pillars of active aging. Exercise is strongly linked to successful aging, with appropriately designed programmes improving outcomes in middle-aged and older adults [6]. It helps maintain cardiorespiratory fitness, muscle strength, and body composition and may slow the progression of chronic disease [7]. Resistance training (RT) is an important nonpharmacological strategy for preserving SMM, strength, and physical function and reducing the risk of sarcopenia in older adults [8,9]. It improves muscle health and delays weakness in older adults [9].

In previously untrained women across middle and later adulthood, RT may also improve muscular performance and support the maintenance of functional capacity. However, RT provides a relatively limited aerobic-training stimulus. Adding endurance exercise to RT may broaden the physiological adaptations achieved through training by targeting both muscular and aerobic outcomes.

Concurrent training (CT) typically combines endurance training and RT within the same programme [10]. Although endurance exercise has sometimes been proposed to attenuate selected RT adaptations through an “interference effect” [11–13], CT may provide broader improvements in cardiorespiratory fitness and metabolic health [14]. In the present study, however, CT consisted of the same RT programme performed by the RT condition followed by additional cycling. Consequently, the randomised training comparison evaluates the effect of adding cycling to RT rather than a comparison of exposure-matched training modalities. The CT condition also involved longer sessions and greater total exercise exposure, which should be considered when interpreting between-condition differences. Few studies have examined the addition of cycling to RT in combination with assigned dietary protein targets in middle-aged to older women, particularly among women with overweight or obesity [15,16].

Dietary protein is an important nutritional factor that may influence adaptations to exercise training. In a previous study of men, we observed similar muscular and body composition adaptations with reported protein intakes of 1.6 and 3.2 g·kg^−1^·d^−1^ during RT and CT [17]. These findings should not be interpreted as demonstrating that 1.6 g·kg^−1^·d^−1^ is universally sufficient or optimal, particularly in middle-aged to older women. Meta-regression evidence from adult RT studies identified a point estimate near 1.6 g·kg^−1^·d^−1^, above which the average additional benefit for fat-free-mass gain appeared to diminish, although substantial uncertainty and individual variability remained [18]. In middle-aged and older adults, protein intake above the Recommended Dietary Allowance (RDA) may support muscular adaptations when combined with exercise, although findings for strength, physical performance, and body composition have been inconsistent [19,20]. Age-related changes in the anabolic response to protein and exercise may also influence dietary requirements, particularly in older adults [21,22]. Protein recommendations of approximately 1.0–1.5 g·kg^−1^·d^−1^ have been proposed for selected older-adult populations, depending on health status, activity level, and clinical context [23,24]. These recommendations should not, however, be applied uniformly to all women aged 40 years and older. In the present trial, 0.8 g·kg^−1^·d^−1^ represented an RDA-level assigned target, whereas 1.6 and 2.2 g·kg^−1^·d^−1^ represented progressively higher assigned targets. Because the intervention used individualised dietary counselling under free-living conditions rather than controlled feeding, these conditions are most appropriately described as assigned protein targets supported by self-reported dietary intake, rather than objectively verified protein diets.

Despite growing interest in combined exercise and nutrition strategies, intervention studies examining training condition × assigned protein-target effects in previously untrained women spanning middle to older age remain limited. To our knowledge, no previous randomised 2 × 3 factorial trial has compared RT with the same RT programme plus cycling (CT) across assigned protein targets of 0.8, 1.6, and 2.2 g·kg^−1^·d^−1^ while assessing SMM, body composition, muscular performance, functional performance, and cycle-derived estimated maximal oxygen uptake (VO₂max) in this population.

Accordingly, the present study examined the training × protein interaction and the marginal effects of randomised training condition and assigned protein target after 12 weeks. SMM was designated as the primary outcome. We hypothesised that, after baseline adjustment, RT would produce higher maximal-strength outcomes than CT, whereas CT would produce higher cycle-derived estimated VO₂max. We further hypothesised that higher assigned protein targets would be associated with more favourable adjusted postintervention muscular outcomes than the 0.8 g·kg^−1^·d^−1^ RDA-level target.

## 2. Materials and methods

### 2.1. Participants

A total of 108 women aged 40–77 years were enroled and randomised in this study. Women were recruited across middle and later adulthood because reductions in SMM, strength, physical function, and cardiometabolic health may begin during midlife and continue with advancing age. This age range was therefore selected to enhance the clinical relevance of the intervention across a broad adult age range. Because menopausal and hormonal status were not formally characterised, the study population was defined according to age rather than presumed menopausal stage. Eligibility was determined through pre-participation health screening based on a structured medical and health-history interview, self-report questionnaires, and investigator review, without laboratory testing or verification against medical records. Individuals were excluded if they reported physician-diagnosed type 2 diabetes, hypertension, cardiovascular disease, clinically relevant sleep disturbances, defined as a self-reported physician-diagnosed sleep disorder, such as insomnia or obstructive sleep apnoea, and/or current use of prescription sleep medication, or another self-reported health condition considered by the screening investigator to contraindicate safe participation in the exercise intervention. Menopausal status was not assessed using a standardised classification system, such as the Stages of Reproductive Aging Workshop criteria, or through hormonal confirmation and was therefore unavailable for covariate adjustment. Any age categories reported in the manuscript were treated as descriptive, nonclinical age bands and were not interpreted as verified premenopausal, perimenopausal, or postmenopausal classifications. Menopausal hormone therapy use was not separately assessed or recorded.

Participants also completed self-report questionnaires regarding health and physical activity. Eligibility required self-reported participation in <2 h/week of structured physical activity during the preceding year. Participants were considered previously untrained based on this activity history; however, resistance-training participation was not assessed using a separate validated physical activity instrument. Participants also reported a habitual sleep duration of approximately 7–8 h/night; this information was used to describe the sample rather than as an eligibility criterion. Sleep quality was additionally assessed using the Pittsburgh Sleep Quality Index (PSQI) to characterise baseline sleep quality, and PSQI scores were not used to determine eligibility. General psychological health was characterised at baseline using the 28-item General Health Questionnaire (GHQ-28). The questionnaire was administered according to its standardised instructions, and the total score was used descriptively; it was not used as an eligibility criterion or included as a covariate.

Participants reported no current use of medications or dietary supplements at enrolment. This information was obtained through general self-report screening; however, because menopausal hormone therapy was not queried separately, its use cannot be assumed to have been absent. The medication and supplement criterion was applied to reduce potential confounding of body composition, muscular-performance, recovery, and dietary-intervention outcomes. Habitual protein intake below approximately 0.8 g·kg^−1^·d^−1^ was an eligibility criterion and was estimated from six self-reported 24-h dietary records collected at baseline. Written informed consent was obtained from all eligible participants after the study procedures had been explained in detail. Participants subsequently received individualised nutritional guidance designed to support assigned protein targets of 0.8, 1.6, or 2.2 g·kg^−1^·d^−1^ under free-living conditions, with protein intake recommended to be distributed across 3–7 daily eating occasions. The study protocol received approval from the Research Ethics Committee of the University of Isfahan (approval code: IR.UI.REC.1404.174) and was conducted in accordance with the Declaration of Helsinki.

### 2.2. Study design

This was a 12-week, single-centre, parallel-group, randomised 2 × 3 factorial trial comparing two active exercise conditions, RT and the same RT programme followed by additional cycling (CT), across three assigned protein targets: 0.8, 1.6, and 2.2 g·kg^−1^·d^−1^. The study did not include a nonexercise, diet-only, or usual-diet control condition. Participants were randomised into one of six groups: RT + 0.8 g·kg^−1^·d^−1^ protein (*n* = 18; RT-0.8), RT + 1.6 g·kg^−1^·d^−1^ protein (*n* = 18; RT-1.6), RT + 2.2 g·kg^−1^·d^−1^ protein (*n* = 18; RT-2.2), CT + 0.8 g·kg^−1^·d^−1^ protein (*n* = 18; CT-0.8), CT + 1.6 g·kg^−1^·d^−1^ protein (*n* = 18; CT-1.6), or CT + 2.2 g·kg^−1^·d^−1^ protein (*n* = 18; CT-2.2). Randomisation was performed using a computer-generated sequence with stratification by baseline SMM to achieve balanced group distributions. Within each SMM stratum, allocation was generated using a restricted randomisation approach (computer-generated permuted blocks) to maintain balance across the six groups; variable block sizes were used and were concealed from investigators involved in recruitment or enrolment. The randomisation sequence was generated by an investigator not involved in recruitment, training supervision, or outcome assessments. Allocation was concealed using sequentially numbered, opaque, sealed envelopes opened after completion of baseline testing. Due to the nature of the exercise interventions, participants and exercise supervisors were not blinded to training modality (RT vs CT). To minimise bias, outcome assessments were performed by assessors blinded to group allocation, and participants were instructed not to disclose their intervention to assessors. Outcome measures were collected at baseline and after 12 weeks at consistent times of day (±1 h). All RT and CT sessions were supervised, scheduled by appointment, and logged to ensure compliance. To improve precision and account for any residual baseline imbalance, baseline values for each outcome were included as covariates in the analysis of covariance (ANCOVA) models.

### 2.3. Body composition

Upon arrival at the laboratory, participants were instructed to void their bladder within 30 minutes before testing to minimise variability related to acute fluid balance. Body mass was measured to the nearest 0.1 kg using a calibrated digital scale (Lumbar, China), and stature was recorded to the nearest 0.1 cm using a wall-mounted stadiometer (Race Industrialisation, China). Body mass index (BMI) was calculated as body mass divided by height squared (kg·m^−2^). Whole-body composition, including multifrequency bioelectrical impedance analysis (BIA)-derived estimates of body fat percentage (BFP) and SMM, was assessed using multifrequency BIA (InBody 270, South Korea) according to the manufacturer’s standardised procedures. Baseline and postintervention assessments were conducted using the same device and standardised testing procedures.

To minimise acute influences on impedance-derived estimates, participants were instructed to fast for ≥12 h, obtain at least 8 h of sleep, and refrain from strenuous exercise and alcohol consumption for 36–48 h before each assessment. Participants were also instructed to maintain a similar food- and fluid-intake routine before their baseline and postintervention assessments. Hydration status was not objectively assessed using urine- or blood-based measures. Menstrual-cycle phase was not standardised, and menopausal status was not determined. The resulting SMM and BFP values were treated as device-derived estimates rather than direct measurements of skeletal muscle or adipose tissue. Although these standardised procedures were intended to reduce acute measurement variability, they could not eliminate the effects of hydration and fluid distribution or the inherent precision limitations of BIA. Study-specific test–retest reliability and minimal detectable change were not established; therefore, small pre–post changes in SMM or BFP should be interpreted cautiously.

### 2.4. Estimated maximal strength assessment

Maximal dynamic strength was estimated using a repetition-based one-repetition maximum (1-RM) prediction procedure rather than directly measured as a true 1-RM for all RT exercises included in the programme, including the plate-loaded leg press and chest press. Estimated 1-RM values for all exercises were used to prescribe individualised RT intensities, whereas the leg-press and chest press values were also analysed as strength outcomes. Before baseline testing, participants completed three familiarisation sessions using the testing equipment and standardised lifting techniques.

At both baseline and postintervention, estimated maximal-strength testing was distributed across three separate sessions rather than completed during a single session to minimise cumulative fatigue. The 11 exercises were divided as follows: session 1, leg press, forward lunge, machine standing calf raise, and back extension; session 2, machine chest press, lat pulldown, seated machine shoulder press, and dumbbell lateral raise; and session 3, machine abdominal crunch, cable biceps curl, and cable triceps pushdown. Within each session, multijoint exercises involving larger muscle groups were assessed before single-joint exercises involving smaller muscle groups. The same exercise allocation and testing order were used at baseline and postintervention. Each testing session lasted approximately 90 min, and consecutive testing sessions were separated by at least 48 h.

Prior to testing, participants were provided with a standardised briefing outlining the purpose of the assessment, potential risks and discomforts, participant responsibilities, potential benefits, and their right to withdraw at any time. To minimise factors that could influence performance, participants were instructed to abstain from alcohol for 48 h, caffeine for 12 h, and food intake for at least 2 h before testing, while water was permitted ad libitum.

Each session began with a 10-min warm-up consisting of 5 min of light aerobic exercise, treadmill walking or jogging at 3–5 km·h^−1^ or elliptical exercise at resistance levels 5–10, followed by 5 min of dynamic mobility exercises (medicine-ball twists, 1 × 10; medicine-ball wood chops, 1 × 10; straddled toe touches, 2 × 5; dynamic quadriceps stretches, 1 × 5; and medicine-ball squats, 1 × 5–8).

Following the standardised warm-up, participants completed 2–3 exercise-specific warm-up sets using progressively heavier loads corresponding to approximately 40%, 60%, and 80% of the anticipated 1-RM. A maximum of two total prediction attempts was permitted for each exercise, with the load selected to elicit technical failure within 2–10 repetitions. A valid attempt required technically correct repetitions performed through the standardised range of motion until the participant could no longer complete another repetition while maintaining the prescribed technique. If more than 10 repetitions were completed during the first attempt, that attempt was treated as a load-selection attempt and was not used to estimate 1-RM. The load was then increased, and a second and final attempt was performed after the prescribed rest interval. If fewer than two repetitions were completed or the prescribed technique or range of motion was not maintained during the first attempt, the load was adjusted before the second and final attempt. If neither attempt met the prespecified validity criteria, no estimated 1-RM value was calculated for that exercise during that testing session.

The load and number of repetitions completed during the heaviest valid attempt were recorded. Rest intervals of 3–5 min were provided between attempts, and 5 min of recovery was provided between exercises, to reduce the influence of fatigue. The testing sequence was identical at baseline and postintervention. Testing was performed using the same equipment at both time points, and assessments were administered by the same assessor following a standardised protocol.

Estimated 1-RM was calculated using the Brzycki equation [25]:

Estimated 1-RM (kg) = load lifted (kg) ÷ [1.0278 − (0.0278 × number of repetitions completed)].

Only attempts completed within the prespecified range of 2–10 repetitions were used in the equation. When both attempts were valid, the heaviest valid attempt was used for calculation. Accordingly, strength values were consistently reported as estimated 1-RM rather than directly measured 1-RM. The resulting estimated 1-RM values were used to determine individualised training intensities for the RT protocols. Given that participants were previously untrained, the relatively low initial training intensities were selected to enhance safety, facilitate technique acquisition, and reduce injury risk during the early phase of the intervention. No study-specific test–retest reliability assessment was performed for the estimated 1-RM procedure.

### 2.5. Muscular endurance

Approximately 5 min after completing the estimated 1-RM assessments, participants performed the leg press and chest press at 50% of their estimated 1-RM to assess relative-load muscular endurance. At baseline, the endurance test load was set at 50% of the baseline estimated 1-RM, whereas at postintervention it was recalculated as 50% of the postintervention estimated 1-RM. The exercise order was standardised as leg press followed by chest press and was kept consistent at baseline and postintervention testing. Muscular endurance was defined as the total number of technically valid repetitions completed to technical failure [26]. Technical failure was defined as the inability to complete another repetition through the prescribed range of motion while maintaining standardised technique. Repetitions were performed at a standardised cadence of 3 s per repetition, controlled using a metronome, and the same cadence was used at baseline and postintervention testing. The same equipment and testing procedures were used at baseline and postintervention, and assessments were administered by the same assessor.

Because muscular endurance testing was performed approximately 5 min after the estimated 1-RM assessments, performance may have been influenced by residual fatigue. In addition, because the endurance test load was derived from the same-day estimated 1-RM, any error in the estimated 1-RM could have propagated to the prescribed 50% load. Furthermore, recalculating the postintervention load from the postintervention estimated 1-RM means that participants generally performed the follow-up endurance test against a different absolute load than at baseline. Accordingly, the results represent relative-load muscular-endurance performance under the standardised testing sequence used in this study rather than fully rested muscular endurance capacity or endurance at an identical absolute load across time points.

### 2.6. Functional performance assessment

Functional performance was assessed using the 30-second chair-stand test for lower-body function and the 30-second arm-curl test for upper-body function, both components of the Senior Fitness Test battery [27]. Before testing, the procedures were demonstrated, and participants completed one familiarisation trial for each test. The tests were administered in a standardised order, with the chair-stand test performed before the arm-curl test, and this order was maintained at baseline and postintervention assessments.

For the chair-stand test, participants sat in the centre of a standard armless chair with a seat height of approximately 43 cm and their arms crossed over the chest. On the start command, participants rose to a fully upright standing position and returned to a fully seated position as many times as possible within 30 s. Only repetitions completed through the required range of motion were counted. The number of technically correct repetitions was recorded as the score. For the arm-curl test, participants sat upright in a chair with their back supported and feet placed flat on the floor, holding a 2.3-kg dumbbell in the dominant hand. The test began with the arm extended alongside the chair and the palm facing the body. Participants flexed the elbow through the prescribed range of motion, rotating the forearm so that the palm faced the shoulder during the upward phase, and then returned the arm to the starting position. Participants performed as many technically correct repetitions as possible within 30 s, and the total number of correct repetitions was recorded.

Two recorded trials were completed for each test, with the higher score used in the analysis. The same chair, dumbbell, equipment setup, instructions, and testing order were used at baseline and postintervention. Assessments were administered by the same trained assessor following a standardised protocol. No study-specific test–retest reliability assessment was performed.

### 2.7. Resistance training

Participants in the three RT groups completed three supervised sessions per week (Saturday, Monday, and Wednesday) under the guidance of certified strength and conditioning specialists. Each session consisted of a full-body RT protocol. A standardised 10-min warm-up, including general and exercise-specific dynamic movements, was performed before training. The exercise programme included the lat pulldown, machine chest press, leg press, forward lunge, machine standing calf raise, dumbbell lateral raise, seated machine shoulder press, machine abdominal crunch, back extension, cable biceps curl, and cable triceps pushdown. The same RT protocol was also performed by participants assigned to the CT conditions before the additional cycling component. During the first 6 weeks, participants performed three sets per exercise, progressing to four sets during the final 6 weeks. Training intensity ranged from 50% to 75% of estimated 1-RM, with prescribed repetition targets ranging from 6 to 16 repetitions per set and interset rest intervals of 30–80 s [28]. Prescribed repetitions were treated as target repetitions rather than mandatory repetitions at a fixed load. When a participant was unable to complete the prescribed repetition target at the calculated relative load while maintaining the required range of motion and proper technique, the supervising staff reduced the load for the subsequent set and/or training session. These adjustments were made to preserve the intended repetition range, maintain training quality, and ensure participant safety. Conversely, loads were increased in accordance with the planned weekly progression when participants completed the prescribed sets and repetitions with correct technique. Rating of perceived exertion (RPE) was monitored using the Borg category-ratio 10 (CR10) scale (0 = no exertion and 10 = maximal exertion) to characterise exercise effort throughout the intervention. Further details of the prescribed weekly progression are presented in Table 1.

**Table 1.** Resistance training protocol.

Week	Intensity (% of estimated 1-RM)	Sets	Repetitions	Rest (seconds)
1	50%	3	16	30
2	50%	3	16	30
3	55%	3	14	40
4	55%	3	14	40
5	60%	3	12	50
6	60%	3	12	50
7	65%	4	10	60
8	65%	4	10	60
9	70%	4	8	70
10	70%	4	8	70
11	75%	4	6	80
12	75%	4	6	80

Values indicate the prescribed weekly progression for the resistance-training component (3 supervised sessions·week^−^^1^). Intensity is expressed as % of estimated 1-RM for each exercise; repetitions are performed per set; rest indicates inter-set rest interval (seconds). 1-RM, one-repetition maximum.

### 2.8. Concurrent training

Participants in the three CT groups also completed three supervised sessions per week (Saturday, Monday, and Wednesday). In each session, participants first completed the identical RT protocol performed by the RT groups, immediately followed by an additional cycling component [17]. Consequently, the CT condition involved longer sessions and greater total exercise exposure than the RT-only condition; total session duration, training work, and exercise-related energy expenditure were not matched between conditions.

The cycling component was performed on a cycle ergometer, with a prescribed duration ranging from 10 to 35 min and an intensity corresponding to 50%–75% of age-predicted maximal heart rate (HRmax = 220 − age). According to the prescribed progression, cycling duration decreased from 35 min at 50% of HRmax during week 1 to 10 min at 75% of HRmax during week 12, while intensity progressively increased. Heart rate was monitored continuously during each cycling session using a Polar chest-strap heart-rate monitor ( Polar Electro Oy, Kempele, Finland), and the cycle-ergometer workload was adjusted when necessary to maintain heart rate near the prescribed target intensity. Further details of the prescribed cycling programme are presented in Table 2.

**Table 2.** Cycling component of the concurrent training condition.

Week	Duration (minutes)	Intensity (% HRmax)
1	35	50%
2	35	50%
3	30	55%
4	30	55%
5	25	60%
6	25	60%
7	20	65%
8	20	65%
9	15	70%
10	15	70%
11	10	75%
12	10	75%

Values indicate the prescribed weekly progression for the additional cycling component of the concurrent training condition. Intensity is expressed as % of age-predicted maximal heart rate (HRmax = 220 − age); duration indicates minutes per session. The RT component was identical to that performed in the RT-alone condition. HRmax, maximal heart rate.

### 2.9. Cycle-derived estimated VO₂max assessment

Participants completed the Ekblom–Bak (EB) submaximal cycle-ergometer test while maintaining a pedal frequency of 60 revolutions per minute (rpm). The full test protocol has been described elsewhere [29,30]. Testing was performed on a mechanically braked Monark 828E cycle ergometer (Monark Exercise AB, Vansbro, Sweden), consistent with the ergometer type used to develop and validate the EB test.

The test began with 4 min of cycling at a standard workload of 0.5 kiloponds (kp), corresponding to approximately 30 W, immediately followed by 4 min at a higher, individually selected submaximal workload. The higher workload was selected based on participants’ self-reported physical activity and fitness levels to elicit an RPE of approximately 14 on the Borg 6–20 scale. The test was discontinued if RPE reached ≥17 [29]. For each participant, the same individually selected higher workload was used at baseline and postintervention testing to standardise the within-participant comparison. The official protocol specifies two consecutive 4-min workloads, an RPE target of approximately 14, and test termination when RPE reaches 17 or higher.

Heart rate, rather than oxygen uptake, was recorded during the final minute of each workload. Heart rate was recorded at 3:15, 3:30, 3:45, and 4:00 min of each workload, and the four values were averaged to represent the mean heart rate for that workload. No respiratory-gas analysis or direct measurement of oxygen uptake was performed.

Estimated VO₂max was calculated using the revised women-specific EB prediction equation [30]:

Estimated VO₂max (L·min^−1^) = exp{(1.84390 − 0.00673 × age) − [0.62578 × (ΔHR/ΔPO)] + (0.00175 × ΔPO) − (0.00471 × HRstandard)}.

Here, exp(x) denotes the natural exponential function, e^x^, where e is approximately 2.71828. Age was entered in years; ΔHR represents the difference in mean heart rate, in beats·min^−1^, between the higher and standard workloads; ΔPO represents the difference in power output, in watts, between the two workloads; and HRstandard represents the mean heart rate, in beats·min^−1^, during the final minute of the standard workload.

The resulting absolute estimated VO₂max value was converted to relative estimated VO₂max as follows:

Relative estimated VO₂max (mL·kg^−1^·min^−1^) = [absolute estimated VO₂max (L·min^−1^) × 1,000] ÷ body mass (kg), using body mass measured at the corresponding baseline or postintervention assessment. The revised equation has demonstrated acceptable validity across a broad range of adult ages and aerobic-fitness levels [30,31]. The official EB protocol uses two consecutive 4-min workloads and bases the estimate on the heart-rate response between them.

To minimise within-participant variability, baseline and postintervention tests were conducted under standardised conditions, including a similar time of day, avoidance of strenuous exercise for 24–48 h, and consistent pretest routines regarding sleep, caffeine, and recent food and fluid intake. The same cycle ergometer, seat and handlebar settings, testing instructions, and heart-rate-monitoring procedures were used at both assessments. Because the EB test is submaximal, the reported values represent cycle-derived estimated VO₂max rather than directly measured maximal oxygen uptake. Changes in submaximal heart-rate response, cycling familiarity, and cycling economy may have influenced the estimated values, particularly because cycling was included only in the CT intervention.

### 2.10. Dietary intervention and assessment

Participants completed six 24-h dietary records, four nonconsecutive weekdays and two nonconsecutive weekend days to estimate baseline habitual energy and macronutrient intake, including protein intake. Participants received standardised instructions regarding the recording of foods, beverages, portion sizes, recipes, and meal timing. A dietary-recording day was considered valid when all foods and beverages consumed during the day were recorded, portion-size information was sufficiently detailed for nutrient coding, and any missing or ambiguous entries were resolved through clarification with the participant. All six valid baseline recording days were required for inclusion in the baseline dietary analysis.

During the 12-week intervention, participants received individualised dietary counselling designed to support assigned protein targets of 0.8, 1.6, or 2.2 g·kg^−1^·d^−1^. Protein targets were calculated using baseline actual body mass and were recalculated weekly using each participant’s most recently measured body mass.

To support target attainment, participants were recommended to consume approximately 20–40 g of protein from animal-based foods, such as chicken breast or comparable sources, after each supervised training session. These foods were self-selected and purchased by participants, and postexercise consumption was recorded by self-report. The remaining daily protein was obtained from self-selected foods of animal and plant origin. Protein sources were not standardised, and the relative contributions of animal- and plant-derived protein were not quantified.

Participants attended consultations with a registered dietitian or nutritionist every 2 weeks and received individualised guidance to meet their assigned protein targets. They were advised to distribute protein across 3–7 daily eating occasions, with approximately 20–40 g of protein per eating occasion [32,33]. Protein distribution and postexercise timing were summarised descriptively from the self-reported dietary records; therefore, these records did not objectively verify that participants followed the recommendations.

Dietary intake, including energy and macronutrients, was monitored throughout the intervention under free-living conditions using participant-completed daily food records entered into MyFitnessPal and additional 24-h dietary records collected every 2 weeks. The records were reviewed regularly by the research team for completeness and plausibility, including missing entries, unusually low or high reported energy intake, and inconsistencies in reported macronutrient totals. Feedback was provided to clarify incomplete or implausible entries and to support attainment of the assigned dietary targets.

An intervention dietary-recording day was excluded when food or beverage intake was incomplete, portion-size information was insufficient for nutrient coding, or unresolved inconsistencies remained after clarification with the participant. Intervention-period dietary means were calculated using all available valid recording days for each participant. Missing or excluded recording days were not replaced, carried forward, or imputed.

All reported dietary-intake data were analysed using Diet Analysis Plus, version 10 (Cengage). Food and beverage entries recorded in MyFitnessPal were manually transferred into Diet Analysis Plus by a member of the research team rather than using the nutrient estimates generated by MyFitnessPal as the final analytical values. When a food was unavailable in the Diet Analysis Plus database, it was coded using the manufacturer’s nutrition information or the closest nutritionally comparable database item. Mixed dishes were separated into their constituent ingredients and entered as recipes. Portion sizes were estimated using household measures, package weights, product labels, and standard serving-size references, as applicable. Records were reviewed for completeness and internal consistency; however, no independent duplicate coding of dietary entries was performed.

The 1.6 g·kg^−1^·d^−1^ target was selected partly based on the meta-regression by Morton et al. [18], which identified a point estimate near which the average additional benefit of higher protein intake for fat-free-mass gain appeared to diminish during RT in adults. This finding was not interpreted as demonstrating that 1.6 g·kg^−1^·d^−1^ is universally sufficient or optimal for middle-aged to older women. The 2.2 g·kg^−1^·d^−1^ target was included to provide clear separation from the 1.6 g·kg^−1^·d^−1^ condition and to examine whether a higher assigned target was associated with additional adaptations. The assigned protein conditions are therefore described using their exact targets rather than qualitative labels such as “low,” “moderate,” or “high.”

Dietary counselling also addressed overall macronutrient composition. Carbohydrate and fat intakes were recommended to fall within the Acceptable Macronutrient Distribution Ranges of 45%–65% and 20%–35% of total energy intake, respectively. Participants were instructed to avoid intentional energy restriction and to consume sufficient energy to support training and recovery.

The dietary conditions were not designed to be isocaloric, and total food intake was not controlled. Increasing protein intake could therefore have altered total energy, carbohydrate, or fat intake. Accordingly, the dietary factor is interpreted as an assigned protein-counselling condition rather than an isolated protein-dose intervention independent of total energy intake.

Dietary intake was assessed using self-reported records. No controlled feeding, direct observation of total daily intake, or objective biomarker of protein consumption was used. The recorded values therefore supported estimated separation among the assigned dietary conditions but did not objectively verify actual intake or adherence.

### 2.11. Statistical analysis

A priori sample-size estimation was performed using G*Power (version 3.1.9.2) within an F-test framework (analysis of covariance [ANCOVA]: fixed effects, main effects, and interactions) for the primary analysis of SMM, with the calculation specifically based on the training-condition × assigned protein-target interaction. The planned factorial design comprised six groups (2 training conditions × 3 assigned protein targets) and included baseline SMM as a covariate. Power was calculated for the training-condition × protein-target interaction (numerator degrees of freedom [df] = 2) using *α* = 0.05, 1 − *β* = 0.80, and a moderate-to-large anticipated effect size (Cohen’s f = 0.35, corresponding to partial η^2^ ≈ 0.11). Under these assumptions, the required total sample size was *N* = 82. To account for anticipated attrition and incomplete pre–post data, 108 participants (*n* = 18 per group) were recruited and randomised. Accordingly, the study was designed to detect a moderate-to-large training-condition × protein-target interaction. Although the final complete-case sample of 83 participants met the prespecified sample-size requirement, the study may have had limited sensitivity to detect smaller protein-target main effects or training-condition × protein-target interactions. Accordingly, nonsignificant findings were not interpreted as evidence of equivalence or absence of an effect.

Data were analysed using SPSS (IBM SPSS Statistics, version 24; IBM Corp., Armonk, NY, USA). The conventional factorial ANCOVA models served as the primary inferential analyses. Model assumptions were evaluated using quantile–quantile (Q–Q) plots of residuals, plots of studentized residuals against fitted values, standardised residuals, Cook’s distance, and Levene’s test. Cook’s distance values greater than 4/N were used as a screening criterion for potentially influential observations but did not automatically result in exclusion. Because selected outcomes showed evidence of non-normal residuals, heteroscedasticity, or influential observations, heteroscedasticity-consistent type 3 (HC3) covariance sensitivity analyses were conducted for all outcomes. All analyses were conducted on a complete-case basis, including 83 participants with complete baseline and week-12 data for every outcome included in the ANCOVA models. No missing outcome values were imputed. Participants included in the analyses were retained in their randomised groups. Because no postintervention outcome data were available for participants who withdrew and no imputation or likelihood-based missing-data method was used, the analyses were complete-case analyses rather than full intention-to-treat estimates. Accordingly, the results represent randomised-condition comparisons among participants with observed follow-up data, and attrition-related bias cannot be excluded.

For each outcome, ANCOVA was performed with the postintervention value as the dependent variable, training condition (RT versus the same RT programme plus cycling [CT]) and assigned protein target (0.8, 1.6, or 2.2 g·kg^−1^·d^−1^) as fixed factors, the training-condition × protein-target interaction as a fixed effect, and the corresponding baseline value as a covariate. For each outcome, the homogeneity-of-regression-slopes assumption was evaluated by testing the interaction between the corresponding baseline value and the six-level randomised-group variable. Evidence of nonparallel baseline slopes was identified for cycle-derived estimated VO₂max and 30-second arm-curl performance. For these outcomes, sensitivity models allowing group-specific baseline slopes were examined, with adjusted training-condition contrasts evaluated at the overall mean of the corresponding baseline outcome.

The interaction effect was evaluated first. When a statistically significant interaction was detected, prespecified simple effects were examined. In the absence of a detectable interaction, interpretation focused on the marginal training-condition and protein-target effects. Planned contrasts included the marginal comparison of CT versus RT, collapsed across assigned protein targets. The omnibus marginal protein-target effect formed part of the factorial model. Pairwise marginal protein-target comparisons, 1.6 versus 0.8, 2.2 versus 0.8, and 2.2 versus 1.6 g·kg^−1^·d^−1^ were treated as exploratory and were collapsed across training condition. Within-training-condition protein comparisons were not interpreted as confirmatory findings when the training-condition × protein-target interaction was not statistically significant. Adjusted marginal means, adjusted mean differences, 95% confidence intervals (CIs), and *p*-values were used to describe the randomised comparisons.

To control for multiplicity, SMM was designated as the primary outcome and tested at *α* = 0.05. For the key secondary outcomes, BFP, leg-press estimated 1-RM, cycle-derived estimated VO₂max, and 30-second chair-stand performance, the four *p*-values from the prespecified marginal training-condition contrasts constituted one multiplicity family and were adjusted using the Holm procedure. Unadjusted and Holm-adjusted *p*-values were reported separately. A separate Holm procedure was applied within each outcome across the three exploratory pairwise protein-target contrasts. Thus, the Holm adjustment for the four key secondary training-condition contrasts and the Holm adjustment for the three within-outcome protein-target contrasts represented two distinct multiplicity families. Unless otherwise specified, the 95% CIs were not adjusted for multiplicity and represent pointwise rather than simultaneous intervals. All other outcomes and comparisons were considered exploratory and interpreted using effect estimates and 95% CIs.

The conventional ANCOVA estimates were retained as the primary results. HC3 sensitivity analyses and group-specific baseline-slope sensitivity analyses were used to evaluate the robustness of the findings and are reported in Supplementary Table S2. Model-sensitive exploratory findings were not treated as confirmatory evidence. Effect sizes for ANCOVA were expressed as partial eta squared (ηp^2^) and interpreted conventionally as small (0.01), medium (0.06), or large (0.14). Post-randomisation energy intake was not included as a covariate in the primary models because it could represent part of, or mediate the effect of, the assigned dietary counselling condition. Differences in reported energy intake were instead considered when interpreting the non-isocaloric dietary conditions. Data are presented as mean ± standard deviation unless otherwise specified, and all statistical tests were two-sided.

## 3. Results

### 3.1. Overview

Baseline characteristics, raw body composition outcomes, raw performance outcomes, factorial ANCOVA results, baseline-adjusted training-condition contrasts, and self-reported dietary intakes are presented in Tables 3–8, respectively. Baseline-adjusted marginal protein-target estimates and exploratory protein-target contrasts are presented in Supplementary Table S1. Raw baseline and week-12 means and percentage changes in Tables 4 and 5 are descriptive; inferential conclusions are based on the baseline-adjusted factorial ANCOVA models. The 95% confidence intervals reported for the planned contrasts are unadjusted pointwise intervals. The conventional factorial ANCOVA models were treated as the primary analyses. Model diagnostics, HC3 heteroscedasticity-consistent analyses, and group-specific baseline-slope sensitivity analyses are presented in Supplementary Table S2. Unless otherwise stated, the inferential results reported below refer to the primary conventional ANCOVA models.

**Table 3.** Baseline characteristics of participants included in the complete-case analyses.

	CT-0.8 (*n* = 14)	CT-1.6 (*n* = 15)	CT-2.2 (*n* = 13)	RT-0.8 (*n* = 13)	RT-1.6 (*n* = 14)	RT-2.2 (*n* = 14)
**Measure**						
**Anthropometry, body composition, health and sleep questionnaires**						
Age (y)	51.5 ± 8.0	46.0 ± 4.7	46.7 ± 5.9	48.5 ± 8.5	49.9 ± 9.7	49.3 ± 6.1
Height (cm)	158.1 ± 4.8	156.8 ± 3.7	157.0 ± 6.3	156.7 ± 5.6	157.8 ± 4.5	159.0 ± 4.9
Body mass (kg)	88.0 ± 15.4	88.6 ± 17.4	83.4 ± 10.3	80.3 ± 15.4	84.1 ± 15.1	83.0 ± 14.6
BMI (kg·m^−2^)	35.1 ± 4.9	36.0 ± 7.2	33.9 ± 4.0	32.9 ± 6.9	33.7 ± 5.5	32.7 ± 4.7
SMM (kg)	25.2 ± 3.4	25.1 ± 3.7	25.2 ± 3.5	24.3 ± 3.2	25.1 ± 3.4	25.4 ± 3.7
BFP (%)	47.3 ± 4.6	47.7 ± 4.3	44.8 ± 5.0	43.6 ± 6.9	44.5 ± 8.5	43.4 ± 6.4
PSQI	3.2 ± 0.8	3.8 ± 1.0	3.5 ± 0.8	3.2 ± 0.5	3.4 ± 0.6	3.6 ± 1.0
GHQ-28	33.4 ± 5.6	37.0 ± 5.3	37.9 ± 6.0	34.3 ± 7.0	38.2 ± 5.3	35.8 ± 6.5
**Age-band distribution (descriptive, nonclinical)**						
40–44 y, n	4	8	4	8	4	4
45–50 y, n	2	6	6	0	7	3
≥51 y, n	8	1	3	5	3	7
**Performance**						
Chest press estimated 1-RM (kg)	29.8 ± 10.3	30.3 ± 9.2	31.4 ± 9.7	29.2 ± 9.7	32.7 ± 13.8	29.3 ± 11.4
Leg press estimated 1-RM (kg)	67.8 ± 17.3	69.6 ± 13.7	70.9 ± 15.8	65.5 ± 13.8	68.7 ± 12.1	69.1 ± 15.0
Chest press endurance (reps)	11.6 ± 1.9	10.7 ± 1.7	10.2 ± 2.1	11.8 ± 1.5	10.7 ± 1.2	11.4 ± 1.6
Leg press endurance (reps)	21.9 ± 2.1	20.8 ± 2.6	23.1 ± 2.1	22.3 ± 2.5	21.4 ± 2.5	23.1 ± 3.1
30-s chair stand (reps)	12.7 ± 3.0	12.6 ± 3.3	14.3 ± 2.9	12.5 ± 3.1	13.2 ± 2.5	14.6 ± 2.9
30-s arm curl (reps)	15.5 ± 4.1	14.6 ± 4.0	15.0 ± 2.9	15.7 ± 4.0	16.4 ± 3.9	16.1 ± 4.4
Cycle-derived estimated VO₂max (mL·kg^−1^·min^−1^)	21.5 ± 4.5	22.0 ± 4.3	21.7 ± 3.9	20.7 ± 3.5	23.5 ± 4.5	22.1 ± 4.1

Values are presented as mean ± standard deviation or n, as appropriate, for the 83 participants with complete baseline and week-12 outcome data. Baseline data for participants who withdrew were unavailable in the final analysis dataset; therefore, the table does not describe the full randomised sample and completers could not be compared with non-completers. Baseline characteristics are presented descriptively without significance testing. The age bands are descriptive, nonclinical categories and do not represent verified menopausal stages; menopausal status and menopausal hormone therapy use were not assessed. Abbreviations: PSQI, Pittsburgh Sleep Quality Index; GHQ-28, General Health Questionnaire-28; BMI, body mass index; BFP, bioelectrical-impedance-analysis-derived body fat percentage; SMM, bioelectrical-impedance-analysis-derived skeletal muscle mass; VO₂max, cycle-derived estimated maximal oxygen uptake. Group definitions: CT-0.8, RT plus cycling with an assigned protein target of 0.8 g·kg^−1^·d^−1^; CT-1.6, RT plus cycling with 1.6 g·kg^−1^·d^−1^; CT-2.2, RT plus cycling with 2.2 g·kg^−1^·d^−1^; RT-0.8, RT alone with 0.8 g·kg^−1^·d^−1^; RT-1.6, RT alone with 1.6 g·kg^−1^·d^−1^; RT-2.2, RT alone with 2.2 g·kg^−1^·d^−1^.

**Table 4.** Raw body composition outcomes at baseline and week 12.

Tier	Outcome	Group	Baseline (mean ± SD)	Week 12 (mean ± SD)	%Δ
Primary	SMM (kg)	CT-0.8	25.16 ± 3.39	25.94 ± 3.28	3.09
		CT-1.6	25.11 ± 3.66	25.97 ± 3.94	3.42
		CT-2.2	25.22 ± 3.45	26.17 ± 3.51	3.75
		RT-0.8	24.33 ± 3.21	25.28 ± 3.21	3.89
		RT-1.6	25.10 ± 3.43	26.22 ± 3.59	4.47
		RT-2.2	25.44 ± 3.68	26.45 ± 3.46	3.96
Key secondary	BFP (%)	CT-0.8	47.32 ± 4.63	46.05 ± 6.16	−2.69
		CT-1.6	47.67 ± 4.27	46.51 ± 6.04	−2.42
		CT-2.2	44.78 ± 5.02	43.84 ± 5.43	−2.1
		RT-0.8	43.55 ± 6.87	44.16 ± 6.93	1.4
		RT-1.6	44.51 ± 8.46	45.24 ± 8.87	1.64
		RT-2.2	43.36 ± 6.37	44.34 ± 6.24	2.27
Secondary	BMI (kg.m^−2^)	CT-0.8	35.05 ± 4.88	35.38 ± 5.33	0.94
		CT-1.6	36.03 ± 7.19	36.65 ± 8.50	1.7
		CT-2.2	33.88 ± 3.96	34.45 ± 4.18	1.7
		RT-0.8	32.87 ± 6.90	34.42 ± 7.14	4.7
		RT-1.6	33.68 ± 5.50	35.54 ± 5.90	5.54
		RT-2.2	32.74 ± 4.71	34.54 ± 4.86	5.5
	Body mass (kg)	CT-0.8	88.04 ± 15.37	88.80 ± 16.01	0.87
		CT-1.6	88.60 ± 17.42	90.01 ± 20.02	1.59
		CT-2.2	83.42 ± 10.29	84.83 ± 10.55	1.7
		RT-0.8	80.32 ± 15.42	84.15 ± 16.06	4.77
		RT-1.6	84.13 ± 15.13	88.77 ± 16.10	5.52
		RT-2.2	82.99 ± 14.61	87.46 ± 14.75	5.39

Values are mean ± standard deviation. Baseline and week-12 values are raw (unadjusted) group means. %Δ = [(week 12 − baseline)/baseline] × 100 and is descriptive only; inferential conclusions are based on the baseline-adjusted factorial ANCOVA models in Tables 6 and 7. SMM and BFP are BIA-derived estimates. Group definitions are provided in Table 3.

**Table 5.** Raw performance outcomes at baseline and week 12.

Tier	Outcome	Group	Baseline (mean ± SD)	Week 12 (mean ± SD)	%Δ
Key secondary	30-s chair stand (reps)	CT-0.8	12.65 ± 3.05	15.79 ± 2.75	24.77
		CT-1.6	12.56 ± 3.31	15.47 ± 2.72	23.13
		CT-2.2	14.29 ± 2.91	16.92 ± 2.60	18.39
		RT-0.8	12.54 ± 3.12	15.69 ± 2.78	25.16
		RT-1.6	13.21 ± 2.46	15.79 ± 2.58	19.48
		RT-2.2	14.57 ± 2.87	16.57 ± 2.50	13.73
	Leg press estimated 1-RM (kg)	CT-0.8	67.85 ± 17.33	73.80 ± 16.82	8.78
		CT-1.6	69.61 ± 13.74	76.21 ± 11.44	9.48
		CT-2.2	70.94 ± 15.77	77.29 ± 13.50	8.96
		RT-0.8	65.53 ± 13.77	79.05 ± 10.68	20.63
		RT-1.6	68.71 ± 12.14	84.11 ± 10.86	22.41
		RT-2.2	69.15 ± 14.96	79.98 ± 12.79	15.67
	Cycle-derived estimated VO₂max (mL·kg^−1^·min^−1^)	CT-0.8	21.47 ± 4.55	27.51 ± 6.23	28.11
		CT-1.6	22.02 ± 4.29	27.77 ± 4.89	26.13
		CT-2.2	21.68 ± 3.93	27.19 ± 5.15	25.37
		RT-0.8	20.67 ± 3.49	22.09 ± 3.42	6.87
		RT-1.6	23.54 ± 4.48	24.88 ± 4.20	5.68
		RT-2.2	22.08 ± 4.09	23.09 ± 3.34	4.57
Secondary	30-s arm curl (reps)	CT-0.8	15.46 ± 4.09	18.00 ± 3.33	16.45
		CT-1.6	14.57 ± 3.95	17.00 ± 2.83	16.68
		CT-2.2	15.03 ± 2.86	17.31 ± 2.53	15.17
		RT-0.8	15.75 ± 3.99	17.77 ± 2.68	12.84
		RT-1.6	16.44 ± 3.88	19.00 ± 3.16	15.58
		RT-2.2	16.06 ± 4.39	18.64 ± 4.01	16.07
	Chest press endurance (reps)	CT-0.8	11.61 ± 1.90	14.00 ± 1.24	20.64
		CT-1.6	10.73 ± 1.67	13.47 ± 1.36	25.47
		CT-2.2	10.18 ± 2.09	12.69 ± 1.89	24.72
		RT-0.8	11.77 ± 1.48	15.23 ± 1.74	29.41
		RT-1.6	10.73 ± 1.23	15.07 ± 1.54	40.4
		RT-2.2	11.45 ± 1.60	15.57 ± 1.95	36.03
	Chest press estimated 1-RM (kg)	CT-0.8	29.83 ± 10.28	35.51 ± 14.45	19.06
		CT-1.6	30.30 ± 9.24	36.05 ± 12.14	18.97
		CT-2.2	31.35 ± 9.75	36.91 ± 12.40	17.72
		RT-0.8	29.17 ± 9.71	39.12 ± 14.00	34.13
		RT-1.6	32.74 ± 13.79	44.44 ± 19.65	35.71
		RT-2.2	29.29 ± 11.44	39.59 ± 15.82	35.18
	Leg press endurance (reps)	CT-0.8	21.94 ± 2.12	23.50 ± 1.91	7.12
		CT-1.6	20.80 ± 2.59	23.20 ± 2.04	11.56
		CT-2.2	23.13 ± 2.06	24.54 ± 1.98	6.09
		RT-0.8	22.28 ± 2.50	25.46 ± 1.71	14.3
		RT-1.6	21.43 ± 2.54	24.50 ± 1.99	14.33
		RT-2.2	23.13 ± 3.12	25.79 ± 2.36	11.5

Values are mean ± standard deviation. Baseline and week-12 values are raw (unadjusted) group means. %Δ = [(week 12 − baseline)/baseline] × 100 and is descriptive only; inferential conclusions are based on the baseline-adjusted factorial ANCOVA models in Tables 6 and 7. Strength outcomes are estimated 1-RM values. VO₂max is a cycle-derived estimate from the submaximal Ekblom–Bak test rather than directly measured maximal oxygen uptake. Group definitions are provided in Table 3.

**Table 6.** Factorial ANCOVA effects for all outcomes (*n* = 83).

Tier	Outcome	Effect	F (df1, df2)	p	ηp^2^
Primary	SMM (kg)	Training condition × protein target	0.155 (2, 76)	0.856	0.004
		Training condition	1.221 (1, 76)	0.273	0.016
		Protein target	0.322 (2, 76)	0.726	0.008
Key secondary	BFP (%)	Training condition × protein target	0.002 (2, 76)	0.998	0.000
		Training condition	20.305 (1, 76)	<0.001	0.211
		Protein target	0.337 (2, 76)	0.715	0.009
Secondary	BMI (kg·m^−2^)	Training condition × protein target	0.010 (2, 76)	0.990	0.000
		Training condition	18.224 (1, 76)	<0.001	0.193
		Protein target	0.330 (2, 76)	0.720	0.009
	Body mass (kg)	Training condition × protein target	0.019 (2, 76)	0.981	0.001
		Training condition	17.334 (1, 76)	<0.001	0.186
		Protein target	0.315 (2, 76)	0.730	0.008
Key secondary	30-s chair stand (reps)	Training condition × protein target	0.724 (2, 76)	0.488	0.019
		Training condition	1.848 (1, 76)	0.178	0.024
		Protein target	2.368 (2, 76)	0.101	0.059
	Leg press estimated 1-RM (kg)	Training condition × protein target	2.564 (2, 76)	0.084	0.063
		Training condition	65.864 (1, 76)	<0.001	0.464
		Protein target	2.763 (2, 76)	0.069	0.068
	Cycle-derived estimated VO₂max (mL·kg^−1^·min^−1^)	Training condition × protein target	0.005 (2, 76)	0.995	0.000
		Training condition	155.943 (1, 76)	<0.001	0.672
		Protein target	0.645 (2, 76)	0.528	0.017
Secondary	30-s arm curl (reps)	Training condition × protein target	3.095 (2, 76)	0.051	0.075
		Training condition	1.237 (1, 76)	0.270	0.016
		Protein target	0.367 (2, 76)	0.694	0.010
	Chest press endurance (reps)	Training condition × protein target	1.123 (2, 76)	0.331	0.029
		Training condition	51.519 (1, 76)	<0.001	0.404
		Protein target	1.188 (2, 76)	0.310	0.030
	Chest press estimated 1-RM (kg)	Training condition × protein target	0.439 (2, 76)	0.646	0.011
		Training condition	136.169 (1, 76)	<0.001	0.642
		Protein target	0.216 (2, 76)	0.807	0.006
	Leg press endurance (reps)	Training condition × protein target	3.087 (2, 76)	0.051	0.075
		Training condition	73.788 (1, 76)	<0.001	0.493
		Protein target	0.630 (2, 76)	0.536	0.016

Factorial ANCOVA modelled the week-12 outcome with the corresponding baseline value as a covariate and included training condition, assigned protein target, and their interaction. Interaction effects are shown first. F-statistics are Type III tests based on the conventional ANCOVA covariance estimator; ηp² is partial eta squared. P-values are unadjusted omnibus values. N = 83 for all models. Model diagnostics and HC3 heteroscedasticity-consistent sensitivity analyses are reported in Supplementary Table S2.

**Table 7.** Baseline-adjusted marginal comparison of CT versus RT.

Category	Tier	Outcome	Adjusted difference (CT − RT)	95% CI	Raw *p*	Holm-adjusted *p*
Body composition	Primary	SMM (kg)	−0.16	−0.45 to 0.13	0.273	—
	Key secondary	BFP (%)	−2.04	−2.94 to −1.14	<0.001	<0.001
	Secondary	BMI (kg·m^−2^)	−1.35	−1.99 to −0.72	<0.001	—
		Body mass (kg)	−3.29	−4.86 to −1.72	<0.001	—
Performance	Key secondary	30-s chair stand (reps)	0.27	−0.13 to 0.67	0.178	0.178
		Leg press estimated 1-RM (kg)	−6.68	−8.32 to −5.04	<0.001	<0.001
		Cycle-derived estimated VO₂max (mL·kg^−1^·min^−1^)	4.53	3.80 to 5.25	<0.001	<0.001
	Secondary	30-s arm curl (reps)	−0.21	−0.59 to 0.17	0.270	—
		Chest press endurance (reps)	−1.53	−1.95 to −1.10	<0.001	—
		Chest press estimated 1-RM (kg)	−5.02	−5.88 to −4.16	<0.001	—
		Leg press endurance (reps)	−1.26	−1.55 to −0.97	<0.001	—

Adjusted differences were calculated as CT minus RT, collapsed across assigned protein targets. Positive values indicate a higher baseline-adjusted mean in CT, whereas negative values indicate a lower baseline-adjusted mean; whether a higher or lower value is favourable depends on the outcome. Raw p-values and 95% CIs are based on the conventional ANCOVA covariance estimator. Holm-adjusted p-values are shown only for the four key secondary outcomes. The 95% CIs are unadjusted pointwise intervals; an em dash indicates that Holm adjustment was not applied. Sensitivity analyses are reported in Supplementary Table S2.

**Table 8.** Self-reported dietary intake at baseline and during the intervention.

Outcome	Group	Baseline (mean ± SD)	Intervention period (mean ± SD)
Energy (kcal·kg^−1^·d^−1^)	CT-0.8	23.47 ± 4.86	23.74 ± 5.31
	CT-1.6	25.58 ± 5.93	28.75 ± 2.97
	CT-2.2	23.40 ± 6.46	29.67 ± 5.10
	RT-0.8	25.56 ± 5.33	27.61 ± 3.67
	RT-1.6	23.72 ± 3.51	27.10 ± 3.30
	RT-2.2	24.49 ± 5.23	30.02 ± 3.30
Protein (g·kg^−1^·d^−1^)	CT-0.8	0.78 ± 0.25	0.86 ± 0.25
	CT-1.6	0.80 ± 0.28	1.58 ± 0.40
	CT-2.2	0.77 ± 0.27	2.17 ± 0.50
	RT-0.8	0.79 ± 0.25	0.85 ± 0.25
	RT-1.6	0.81 ± 0.28	1.62 ± 0.40
	RT-2.2	0.78 ± 0.28	2.21 ± 0.50
Carbohydrate (g·kg^−1^·d^−1^)	CT-0.8	3.85 ± 1.30	3.80 ± 1.12
	CT-1.6	4.15 ± 1.13	4.19 ± 0.62
	CT-2.2	3.52 ± 1.32	3.75 ± 0.73
	RT-0.8	4.16 ± 1.05	4.56 ± 0.94
	RT-1.6	3.93 ± 0.92	3.97 ± 0.61
	RT-2.2	3.95 ± 1.27	3.95 ± 0.83
Fat (g·kg^−1^·d^−1^)	CT-0.8	0.55 ± 0.17	0.57 ± 0.15
	CT-1.6	0.64 ± 0.22	0.63 ± 0.12
	CT-2.2	0.70 ± 0.25	0.66 ± 0.21
	RT-0.8	0.64 ± 0.16	0.66 ± 0.18
	RT-1.6	0.53 ± 0.20	0.53 ± 0.13
	RT-2.2	0.62 ± 0.14	0.60 ± 0.13

Values are group means ± sample standard deviations calculated across the available participant-level values derived from self-reported dietary records; the reported standard deviations represent between-participant variability and are not standard errors. Baseline and intervention-period intakes are shown; the intervention-period values are not a single post-intervention measurement. For each participant, relative energy intake was calculated from protein, carbohydrate, and fat intakes using energetic coefficients of 4, 4, and 9 kcal·g^−1^, respectively, after which group means and sample standard deviations were calculated. Energy is expressed as kcal·kg^−1^·d^−1^ and macronutrients as g·kg^−1^·d^−1^. The dietary conditions were free-living and non-isocaloric, and no controlled feeding or objective biomarker of protein intake was used; therefore, the values support reported separation among assigned targets but do not objectively verify intake or adherence. Group definitions are provided in Table 3.

### 3.2. Participant characteristics

One hundred fifty participants were assessed for eligibility. Thirty did not meet the inclusion criteria, and 12 declined to participate after the initial interview. Accordingly, 108 participants were randomised into the six conditions (*n* = 18 per condition). During the intervention, four participants withdrew from CT-0.8, three from CT-1.6, five from CT-2.2, five from RT-0.8, four from RT-1.6, and four from RT-2.2. Withdrawals were attributed to time constraints or loss of interest. Overall, 25 of 108 randomised participants withdrew, corresponding to an attrition rate of 23.1%. The final complete-case sample included 83 participants: CT-0.8 (*n* = 14), CT-1.6 (*n* = 15), CT-2.2 (*n* = 13), RT-0.8 (*n* = 13), RT-1.6 (*n* = 14), and RT-2.2 (*n* = 14). Thus, 83 of 108 randomised participants (76.9%) completed week-12 testing and were included in all outcome analyses. Among the 108 randomised participants, no intervention-related adverse events were reported. Baseline characteristics of the 83 participants included in the complete-case analyses are presented descriptively in Table 3. Baseline characteristics of participants who withdrew were unavailable in the final analytic dataset; therefore, differences between completers and noncompleters at enrolment could not be evaluated. Menopausal status and menopausal hormone therapy use were not assessed. The categories presented in Table 3 are descriptive, nonclinical age bands of 40–44, 45–50, and ≥51 years and should not be interpreted as verified premenopausal, perimenopausal, or postmenopausal classifications.

### 3.3. Body composition

Raw baseline and week-12 BIA-derived body composition estimates are presented in Table 4.

#### 3.3.1. Skeletal muscle mass: primary outcome

The training-condition × protein-target interaction for baseline-adjusted week-12 SMM was not statistically significant, F(2, 76) = 0.16, *p* = 0.856, ηp^2^ = 0.004. No detectable marginal protein-target effect was observed, F(2, 76) = 0.32, *p* = 0.726, ηp^2^ = 0.008, and no detectable marginal training-condition effect was observed, F(1, 76) = 1.22, *p* = 0.273, ηp^2^ = 0.016 (Table 6). The adjusted difference between CT and RT was −0.16 kg (95% CI, −0.45 to 0.13; *p* = 0.273), indicating no detectable between-condition difference in week-12 SMM (Table 7). The raw increases shown in Table 4 are descriptive and should not be interpreted as confirmed hypertrophy or as causal within-condition effects.

#### 3.3.2. Body fat percentage: key secondary outcome

No training-condition × protein-target interaction was detected for baseline-adjusted week-12 BFP, F(2, 76) = 0.002, *p* = 0.998, ηp^2^ < 0.001, and there was no detectable marginal protein-target effect, F(2, 76) = 0.34, *p* = 0.715, ηp^2^ = 0.009. A marginal training-condition effect was observed, F(1, 76) = 20.31, *p* < 0.001, ηp^2^ = 0.211 (Table 6). CT had a lower baseline-adjusted week-12 BFP than RT, with an adjusted difference of −2.04 percentage points (95% CI, −2.94 to −1.14; raw *p* < 0.001; Holm-adjusted *p* < 0.001) (Table 7). This estimate represents a baseline-adjusted comparison between the two active training conditions and should not be described as direct evidence of fat loss or as an effect of cycling independent of the additional exercise exposure.

#### 3.3.3. Secondary body composition outcomes

For BMI, no training-condition × protein-target interaction was detected (*p* = 0.990), and there was no detectable marginal protein-target effect (*p* = 0.720). A marginal training-condition effect was observed (*p* < 0.001, ηp^2^ = 0.193), with a lower baseline-adjusted week-12 BMI in CT than in RT (adjusted difference, −1.35 kg·m^−2^; 95% CI, −1.99 to −0.72; *p* < 0.001) (Tables 6 and 7). For body mass, no training-condition × protein-target interaction was detected (*p* = 0.981), and there was no detectable marginal protein-target effect (*p* = 0.730). A marginal training-condition effect was observed (*p* < 0.001, ηp^2^ = 0.186), with a lower baseline-adjusted week-12 body mass in CT than in RT (adjusted difference, −3.29 kg; 95% CI, −4.86 to −1.72; *p* < 0.001) (Tables 6 and 7). These estimates represent baseline-adjusted week-12 differences between active conditions rather than evidence of absolute weight loss. As shown in Table 4, the raw mean body mass and BMI values increased numerically from baseline in all six conditions.

### 3.4. Performance outcomes

Raw baseline and week-12 performance outcomes are presented descriptively in Table 5.

#### 3.4.1. Leg-press estimated 1-RM: key secondary outcome

In the primary conventional ANCOVA, the training-condition × protein-target interaction was not statistically significant for baseline-adjusted leg-press estimated 1-RM, F(2, 76) = 2.56, *p* = 0.084, ηp^2^ = 0.063. The marginal protein-target effect was also not statistically significant, F(2, 76) = 2.76, *p* = 0.069, ηp^2^ = 0.068. A marginal training-condition effect was observed, F(1, 76) = 65.86, *p* < 0.001, ηp^2^ = 0.464 (Table 6). CT had a lower baseline-adjusted week-12 leg-press estimated 1-RM than RT (adjusted difference, −6.68 kg; 95% CI, −8.32 to −5.04; raw *p* < 0.001; Holm-adjusted *p* < 0.001) (Table 7). This contrast supports a difference between the randomised training conditions but does not establish that cycling itself caused an interference effect.

#### 3.4.2. Cycle-derived estimated VO₂max: key secondary outcome

No training-condition × protein-target interaction was detected for cycle-derived estimated VO₂max, F(2, 76) = 0.005, *p* = 0.995, ηp^2^ < 0.001, and there was no detectable marginal protein-target effect, F(2, 76) = 0.65, *p* = 0.528, ηp^2^ = 0.017. A marginal training-condition effect was observed, F(1, 76) = 155.94, *p* < 0.001, ηp^2^ = 0.672 (Table 6). CT had a higher baseline-adjusted week-12 cycle-derived estimated VO₂max than RT (adjusted difference, 4.53 mL·kg^−1^·min^−1^; 95% CI, 3.80 to 5.25; raw *p* < 0.001; Holm-adjusted *p* < 0.001) (Table 7). The outcome represents an estimate derived from a submaximal cycling test rather than directly measured oxygen consumption or a direct measurement of cardiovascular adaptation.

#### 3.4.3. Thirty-second chair stand: key secondary outcome

In the primary conventional ANCOVA, no detectable training-condition × protein-target interaction was observed for chair-stand performance, F(2, 76) = 0.72, *p* = 0.488, ηp^2^ = 0.019. Neither the marginal protein-target effect, F(2, 76) = 2.37, *p* = 0.101, ηp^2^ = 0.059, nor the marginal training-condition effect, F(1, 76) = 1.85, *p* = 0.178, ηp^2^ = 0.024, was statistically significant (Table 6). The adjusted difference between CT and RT was 0.27 repetitions (95% CI, −0.13 to 0.67; raw *p* = 0.178; Holm-adjusted *p* = 0.178) (Table 7), indicating no detectable between-condition difference. The raw pre–post increases in Table 5 should not be interpreted as randomised evidence that either active intervention improved functional performance.

### 3.5. Secondary performance outcomes

#### 3.5.1. Chest press estimated 1-RM

No training-condition × protein-target interaction (*p* = 0.646) or marginal protein-target effect (*p* = 0.807) was detected. A marginal training-condition effect was observed (*p* < 0.001, ηp^2^ = 0.642), with a lower baseline-adjusted week-12 chest press estimated 1-RM in CT than in RT (adjusted difference, −5.02 kg; 95% CI, −5.88 to −4.16; *p* < 0.001) (Tables 6 and 7).

#### 3.5.2. Chest press endurance

No training-condition × protein-target interaction (*p* = 0.331) or marginal protein-target effect (*p* = 0.310) was detected. A marginal training-condition effect was observed (*p* < 0.001, ηp^2^ = 0.404), with lower baseline-adjusted week-12 chest press endurance in CT than in RT (adjusted difference, −1.53 repetitions; 95% CI, −1.95 to −1.10; *p* < 0.001) (Tables 6 and 7).

#### 3.5.3. Leg press endurance

The training-condition × protein-target interaction was not statistically significant, F(2, 76) = 3.09, *p* = 0.051, ηp^2^ = 0.075. There was no detectable marginal protein-target effect (*p* = 0.536), whereas a marginal training-condition effect was observed (*p* < 0.001, ηp^2^ = 0.493). CT had lower baseline-adjusted week-12 leg-press endurance than RT (adjusted difference, −1.26 repetitions; 95% CI, −1.55 to −0.97; *p* < 0.001) (Tables 6 and 7).

#### 3.5.4. Thirty-second arm curl

In the primary conventional ANCOVA, the training-condition × protein-target interaction was not statistically significant, F(2, 76) = 3.10, *p* = 0.051, ηp^2^ = 0.075. Neither the marginal protein-target effect (*p* = 0.694) nor the marginal training-condition effect (*p* = 0.270, ηp^2^ = 0.016) was statistically significant. The adjusted difference between CT and RT was −0.21 repetitions (95% CI, −0.59 to 0.17; *p* = 0.270) (Tables 6 and 7). The interaction estimates for arm-curl performance and leg-press endurance were F = 3.095 and F = 3.087, respectively, before rounding.

### 3.6. Marginal protein-target estimates

In the primary conventional ANCOVA models, no statistically significant omnibus marginal protein-target effect was detected for any body composition or performance outcome; omnibus *p*-values ranged from 0.069 to 0.807 (Table 6 and Supplementary Table S1). Exploratory marginal protein-target contrasts, collapsed across training condition, identified two nominal raw *p*-values below 0.05. The 2.2 versus 0.8 g·kg^−1^·d^−1^ contrast for chair-stand performance was −0.54 repetitions (95% CI, −1.05 to −0.03; raw *p* = 0.037; Holm-adjusted *p* = 0.111), and the 2.2 versus 1.6 g·kg^−1^·d^−1^ contrast for leg-press estimated 1-RM was −2.26 kg (95% CI, −4.25 to −0.27; raw *p* = 0.027; Holm-adjusted *p* = 0.081). Neither contrast remained statistically significant after adjustment across the three pairwise protein-target comparisons for the relevant outcome. Because no training-condition × protein-target interaction was statistically significant in the primary conventional models, six-condition rankings and within-training-condition protein comparisons were not interpreted as confirmatory findings.

### 3.7. Sensitivity analyses

Model diagnostics indicated departures from residual normality for SMM, BFP, and chair-stand performance and evidence of unequal residual variance for chair-stand performance, leg-press estimated 1-RM, and cycle-derived estimated VO₂max. Evidence of nonparallel baseline slopes was identified for cycle-derived estimated VO₂max and 30-second arm-curl performance (Supplementary Table S2). HC3 heteroscedasticity-consistent analyses did not materially alter the primary SMM conclusion or the principal training-condition findings. However, selected exploratory protein-target and interaction findings were sensitive to covariance specification. The HC3 marginal protein-target *p*-values were 0.048 for chair-stand performance and 0.040 for leg-press estimated 1-RM, whereas the HC3 training-condition × protein-target interaction *p*-value for arm-curl performance was 0.049. These sensitivity findings were exploratory and were not interpreted as confirmatory evidence. In sensitivity models allowing group-specific baseline slopes, CT remained associated with a higher cycle-derived estimated VO₂max than RT (adjusted difference, 4.62 mL·kg^−1^·min^−1^; 95% CI, 3.98 to 5.26; *p* < 0.001). The corresponding adjusted training-condition difference for arm-curl performance remained nondetectable (−0.22 repetitions; 95% CI, −0.57 to 0.14; *p* = 0.232). Exploratory HC3 pairwise protein-target analyses showed an adjusted difference of −0.54 repetitions for the 2.2 versus 0.8 g·kg^−1^·d^−1^ chair-stand comparison (95% CI, −1.02 to −0.06; raw *p* = 0.028; Holm-adjusted *p* = 0.085) and −2.26 kg for the 2.2 versus 1.6 g·kg^−1^·d^−1^ leg-press estimated 1-RM comparison (95% CI, −4.07 to −0.45; raw *p* = 0.015; Holm-adjusted *p* = 0.046). Because these results arose from exploratory sensitivity analyses and differed from the conventional covariance results, they should be interpreted cautiously.

### 3.8. Self-reported dietary intake and nutrient estimates

Self-reported dietary intake is summarised in Table 8. During the intervention, mean reported protein intake approximated the assigned targets and showed separation in the recorded values across the three dietary conditions within both training conditions: 0.8 g·kg^−1^·d^−1^ (CT-0.8: 0.86 ± 0.25; RT-0.8: 0.85 ± 0.25), 1.6 g·kg^−1^·d^−1^ (CT-1.6: 1.58 ± 0.40; RT-1.6: 1.62 ± 0.40), and 2.2 g·kg^−1^·d^−1^ (CT-2.2: 2.17 ± 0.50; RT-2.2: 2.21 ± 0.50). Relative energy intake, calculated at the participant level from protein, carbohydrate, and fat intakes using energetic coefficients of 4, 4, and 9 kcal·g^−1^, respectively, increased numerically from baseline to the intervention period in all six conditions, although the magnitude of the increase varied across conditions. Reported carbohydrate and fat intakes showed mixed and generally smaller descriptive changes. No inferential dietary comparisons were performed.

## 4. Discussion

Across several outcomes, the randomised training-condition contrasts were larger and more precisely estimated than the protein-target contrasts. Compared with RT, CT was associated with lower baseline-adjusted week-12 BFP, BMI, and body mass and with higher cycle-derived estimated VO₂max, whereas the RT condition yielded higher adjusted resistance-test strength and muscular-endurance performance. No detectable training-condition differences were observed for SMM, chair-stand performance, or arm-curl performance. In the primary conventional ANCOVA models, no training-condition × protein-target interactions or omnibus marginal protein-target effects were detected. Therefore, the findings should not be interpreted as showing that exercise “modality” was the dominant biological driver of adaptation; rather, they describe differences between two active intervention packages, one consisting of RT and the other consisting of the same RT programme plus additional cycling (CT) and greater total exercise exposure.

The absence of detectable protein-target effects should also be interpreted cautiously. Although the complete-case sample met the prespecified requirement for detecting a moderate-to-large training-condition × protein-target interaction, the study may have had limited sensitivity to smaller marginal protein effects or interactions. In addition, selected exploratory HC3 sensitivity analyses produced model-sensitive findings for chair-stand performance, leg-press estimated 1-RM, and arm-curl performance; these findings were not treated as confirmatory. Dietary exposure was estimated using self-reported records under free-living, non-isocaloric conditions, without controlled feeding or an objective biomarker of protein intake. Accordingly, the protein-related findings represent comparisons among assigned dietary-counselling conditions supported by reported intake rather than comparisons among objectively verified, isolated protein doses. The absence of detectable differences should not be interpreted as evidence of equivalence, proof that 0.8 g·kg^−1^·d^−1^ was sufficient, or evidence that dietary protein is unimportant for training adaptations. These conclusions apply specifically to the analysed completers in this selected sample of previously untrained women aged 40–77 years.

### 4.1. Skeletal muscle mass

All groups showed small raw increases in SMM of approximately 3%–5%, but baseline-adjusted week-12 SMM did not differ by training condition or assigned protein target. Because the study lacked a nonexercise comparator, these raw increases cannot be attributed solely to the interventions and may also reflect repeated measurement, temporal variability, regression to the mean, or BIA measurement error. Furthermore, SMM is an indirect estimate that is sensitive to hydration and should not be interpreted as direct evidence of skeletal-muscle hypertrophy.

The findings therefore do not establish that the RT component was “sufficient” to stimulate muscle growth. The supported interpretation is that small raw increases in estimated SMM occurred across the active intervention conditions, whereas no detectable baseline-adjusted differences were observed between RT and CT or among the assigned protein targets. Because participants were previously untrained, their responses to a novel exercise programme may have reduced the detectability of smaller incremental differences among the dietary conditions. However, previous training status was not experimentally compared and cannot be identified as the cause of the null protein-target findings. The conservative initial loading, gradual progression, and need for technique acquisition may also have limited the magnitude of the hypertrophic stimulus. These factors may have been appropriate for safety and familiarisation but could have reduced sensitivity to small between-condition differences in SMM. Early adaptations in previously untrained participants may include improvements in lifting technique, motor learning, and neural function, particularly for strength outcomes. Because neural or technical adaptations were not directly measured, they should be regarded only as plausible literature-based explanations rather than established mechanisms in the present study.

Previous studies indicate that RT can increase muscle size in adult women and that training volume, programme duration, and loading progression may influence the magnitude of adaptation [34–36]. However, the literature does not support describing 12 weeks as universally “ideal” for muscle growth, because the time course and magnitude of hypertrophy vary according to population characteristics, training dose, adherence, measurement method, and previous training experience. Although small raw SMM increases were observed in the CT conditions, the baseline-adjusted SMM comparison between CT and RT was not statistically significant. Thus, no detectable interference-type pattern was observed for SMM. The absence of a difference should not be interpreted as evidence that the two conditions were equivalent. The RT component was performed before cycling in the CT condition. Exercise order, recovery, session duration, and total training exposure have been proposed to influence concurrent-training adaptations [37–39]. However, the present design cannot isolate the independent effect of cycling sequence or identify a specific interference mechanism because total exercise duration, work, and energy expenditure were not matched and the proposed mechanisms were not measured.

The higher assigned protein targets did not produce detectable differences in baseline-adjusted SMM in the primary models. Previous literature suggests that postexercise protein ingestion may support adaptations to RT and could theoretically influence CT responses [17,40]. Nevertheless, the current findings indicate only that no detectable differences were observed among the assigned dietary-counselling conditions during this 12-week intervention. They do not demonstrate that objectively verified intakes above 0.8 g·kg^−1^·d^−1^ provide no benefit or that 0.8 g·kg^−1^·d^−1^ is adequate for every participant.

Several factors may have limited the detection of protein-related differences, including the modest hypertrophic stimulus, intervention duration, BIA precision, interindividual variability, uncertainty in self-reported dietary intake, non-isocaloric dietary exposure, and limited sensitivity to small effects. Age-related anabolic resistance, hormonal status, muscle-protein synthesis, leucine availability, inflammatory signalling, and anabolic pathways were not measured. Menopausal status and menopausal hormone therapy use were also not characterised. These mechanisms therefore cannot be used as established explanations for the observed findings.

### 4.2. Body fat percentage

CT was associated with a lower baseline-adjusted week-12 BFP than RT. This result represents an adjusted comparison between two active intervention packages and should not be described as proof that CT caused fat loss or that cycling alone produced the difference. The CT condition included the same RT programme plus additional cycling, longer sessions, and likely greater exercise-related energy expenditure.

The raw BFP values decreased numerically in the CT groups and increased numerically in the RT groups; however, these raw within-condition changes are descriptive. The primary inference is the baseline-adjusted between-condition contrast. Furthermore, because BFP was estimated using BIA, relatively small changes should be interpreted with consideration of hydration sensitivity and measurement precision.

No detectable marginal protein-target effect or training-condition × protein-target interaction was observed for BFP in the primary conventional model. Accordingly, apparent numerical differences among the six intervention cells should not be interpreted as evidence that any specific protein target was superior for reducing BFP. Previous studies examining protein intake, RT, and CT have reported mixed body composition findings [41,42]. Differences in sex, age, training experience, intervention duration, total exercise exposure, energy intake, dietary control, and body composition methodology limit direct comparison with the present study. The present findings do not establish that protein intake has no role in body-fat regulation. Rather, they indicate that no detectable BFP differences were observed among the assigned protein-target conditions during the study. Because total dietary intake was not controlled and reported energy intake differed across conditions, the dietary intervention cannot be interpreted as an isolated protein effect independent of energy exposure.

Overall, CT yielded a lower baseline-adjusted week-12 BFP than RT, but this contrast should be interpreted as a comparison between intervention packages with unequal total exercise exposure.

### 4.3. Lower-body and upper-body strength and muscular endurance

The RT condition yielded higher baseline-adjusted week-12 leg-press and chest press estimated 1-RM values than the CT condition. This pattern is consistent with, but does not prove, an interference-type effect. The design did not directly measure molecular or neuromuscular interference mechanisms and did not match total exercise duration, work, fatigue, or energy expenditure between training conditions. Alternative explanations include resistance-test specificity, greater accumulated fatigue in the CT condition, differences in recovery demands, and unmeasured variation in achieved training dose. Therefore, the findings should be described as higher adjusted resistance-test performance following RT rather than as definitive evidence that cycling physiologically inhibited strength development.

Raw strength values increased numerically in all six conditions. Because there was no inactive comparator, these raw increases cannot be interpreted as causal within-condition improvements. The randomised inference is that RT produced higher baseline-adjusted week-12 resistance-test performance than the same RT programme followed by cycling. The assigned protein target did not produce a detectable omnibus marginal effect on strength in the primary conventional models, and no statistically significant training-condition × protein-target interaction was observed. However, the leg-press protein-target result was sensitive to the covariance specification in exploratory HC3 analyses. Consequently, the protein findings should not be summarised categorically as showing “no effect” under all analytical specifications. Early strength adaptation is often discussed in relation to motor learning, improved coordination, motor-unit recruitment, firing frequency, and reduced antagonist coactivation [43,44]. None of these outcomes was measured in the present study. They may be mentioned as possible literature-based explanations for why resistance-test performance can change more rapidly than muscle size, but they should not be presented as mechanisms demonstrated by the current data. Previous research indicates that RT can improve resistance-test strength in middle-aged and older women [35,36]. Concurrent-training studies have produced mixed findings regarding whether the addition of endurance exercise attenuates strength development [12,45]. The present findings add to this literature by showing higher adjusted resistance-test performance in RT than in CT, while leaving the underlying mechanism unresolved. The primary conventional analyses did not detect protein-target effects on maximal strength or muscular endurance. Previous studies have similarly reported limited short-term effects of additional protein on strength outcomes under some conditions [17,42,46]. Nevertheless, these findings should not be explained by claiming that habitual intake was “adequate,” because individual adequacy was not established and actual intake was not objectively verified. The appropriate interpretation is that no consistent additional strength or muscular-endurance advantage was detected among the assigned protein-target conditions in the primary models. The findings do not establish that verified increases from 0.8 to 1.6 or 2.2 g·kg^−1^·d^−1^ cannot affect neuromuscular adaptation in other populations, over longer interventions, or under controlled dietary conditions. RT also yielded higher baseline-adjusted chest press and leg-press endurance than CT. This pattern may partly reflect exercise specificity because the endurance tests used the same resistance exercises included in the training programme. Residual fatigue, recovery demands, achieved training volume, and the additional cycling exposure may also have contributed. The physiological basis of the difference cannot be determined from the current data.

### 4.4. Functional performance

No detectable baseline-adjusted differences were observed between RT and CT for the 30-second chair-stand or arm-curl tests in the primary conventional models. No consistent marginal protein-target effects or training-condition × protein-target interactions were identified. Raw chair-stand and arm-curl scores increased numerically across the active conditions. However, because the study lacked a nonexercise comparator, these raw increases cannot establish that either RT or CT caused improvements in functional capacity. They may also reflect familiarisation, repeated testing, regression to the mean, natural variation, or nonspecific study participation effects. Accordingly, the findings should not be summarised as showing that both RT and CT improved functional capacity. The supported conclusion is that no detectable randomised between-condition differences were observed in these functional tests over 12 weeks. Previous systematic reviews and trials indicate that RT and multicomponent exercise programmes can improve functional performance in older adults [47–52]. These studies provide contextual support for the broader value of exercise but do not establish that the raw changes observed in the present study were caused by the interventions. Differences in age, frailty, residential setting, baseline function, intervention dose, and comparator groups also limit direct comparison. Proposed explanations involving neuromuscular control, motor learning, cardiovascular adaptation, reduced fatigue, or fall-risk reduction were not assessed and should not be presented as mechanisms demonstrated in this study. Studies examining higher protein intake and functional performance have reported mixed findings [20,53]. In the present study, no consistent functional advantage was detected among the higher assigned protein-target conditions. However, the chair-stand protein-target result was sensitive to the covariance estimator in exploratory analyses. Combined with the uncertainty of self-reported intake and the relatively short intervention duration, this precludes firm conclusions about objectively verified protein intake and longer-term functional adaptation.

### 4.5. Cardiorespiratory fitness (estimated VO₂max)

CT yielded a substantially higher baseline-adjusted week-12 cycle-derived estimated VO₂max than RT. This finding is consistent with the inclusion of repeated cycling in the CT condition and the use of a cycle-based submaximal fitness test. The outcome was not directly measured maximal oxygen uptake. No respiratory-gas analysis was performed, and the study did not directly measure stroke volume, cardiac output, capillary density, mitochondrial enzymes, oxygen extraction, or other central or peripheral cardiovascular adaptations. Therefore, the result should not be described as CT being more effective at improving oxygen consumption or as direct evidence of cardiovascular adaptation. The Ekblom–Bak test estimates VO₂max from the heart-rate response to submaximal cycling workloads. Changes in submaximal heart rate, cycling familiarity, pedalling economy, and test-specific adaptation may therefore have contributed to the higher estimated values in the CT condition. This is particularly relevant because only the CT participants performed regular cycle training. Previous research indicates that endurance and CT can increase directly measured or estimated aerobic-fitness outcomes in middle-aged and older adults [54–62]. Some RT studies have also reported improvements in aerobic-fitness measures [54,61,62]. However, variation in testing method, training mode, age, health status, and whether oxygen uptake was measured directly limits comparison across studies. Central and peripheral adaptations such as improved oxygen delivery, extraction, capillary density, mitochondrial enzyme activity, and muscle-fibre recruitment are plausible mechanisms described in the literature [63], but they were not measured in the present study and should be framed only as hypotheses. No detectable marginal protein-target effect or training-condition × protein-target interaction was observed for cycle-derived estimated VO₂max in the primary models. Thus, the findings indicate that the assigned dietary-counselling conditions did not detectably modify the estimated aerobic-fitness response. Because intake was self-reported and dietary conditions were not isocaloric, the results should not be interpreted as confirming the absence of a biological effect of objectively verified protein intake. Overall, the CT condition produced a higher cycle-derived estimated VO₂max than RT, but the result should be interpreted in the context of additional cycling exposure, cycle-specific testing, and the indirect nature of the prediction method.

### 4.6. Limitations

Body composition was assessed using multifrequency BIA rather than dual-energy X-ray absorptiometry (DXA) or a direct imaging-based assessment of muscle size. Although standardised preassessment procedures were used to reduce variability related to fasting status, recent exercise, alcohol intake, sleep, bladder emptying, and acute hydration, BIA remains sensitive to fluid distribution and provides indirect, device-derived estimates of SMM and BFP. Because the observed SMM changes were small, measurement variability may have limited the ability to distinguish biological change from measurement error and reduced sensitivity to small between-condition differences. Accordingly, the SMM findings should be interpreted as changes in BIA-derived estimates rather than direct evidence of skeletal-muscle hypertrophy, and nonsignificant differences should not be interpreted as evidence of equivalence. No direct muscle-imaging method or study-specific estimate of BIA test–retest reliability or minimal detectable change was available.

The study did not include a nonexercise control, diet-only control, or usual-diet control condition. Consequently, the raw pre–post changes observed within the six active intervention conditions cannot be separated from repeated testing, familiarisation, regression to the mean, temporal variation, natural variability, or other nonspecific study effects. Causal interpretation should therefore be restricted to the randomised comparisons among the active training and dietary conditions. The absence of diet-only and usual-diet comparators also prevents isolation of the effects of dietary counselling or assigned protein targets in the absence of exercise.

Dietary intake and reported adherence to the assigned protein targets were assessed primarily using self-reported food records and repeated 24-h dietary records. Despite regular review by the research team, dietary counselling, plausibility checks, and use of a consistent nutrient-analysis database, these methods remain susceptible to underreporting, overreporting, inaccurate portion-size estimation, dietary-recording reactivity, reporting influenced by knowledge of the assigned targets, and food-database error. The intervention-period standard deviations represent between-participant variability in participant-level mean reported intake and do not capture within-participant day-to-day variability. Nevertheless, the close correspondence between the reported group means and assigned targets should be interpreted cautiously because the dietary data were self-reported and may have been influenced by intensive dietary counselling, repeated monitoring, dietary-recording practices, knowledge of the assigned targets, portion-size estimation error, and food-database limitations rather than objectively verified intake.

Because no objective biomarker of protein intake or controlled-feeding procedure was available, the extent to which the recorded values represented habitual intake throughout the intervention cannot be confirmed. Accordingly, the apparent separation among the recorded protein conditions should not be interpreted as objective confirmation that participants consistently consumed the exact assigned amounts.

The dietary conditions were not designed to be isocaloric, and reported energy intake increased numerically from baseline in all six conditions, with the magnitude of increase differing among conditions. Increasing the assigned protein target may therefore also have altered total energy, carbohydrate, fat, and food intake. The dietary factor should consequently be interpreted as an assigned protein-counselling package rather than as an isolated protein-dose effect independent of energy exposure.

Protein targets were calculated using actual body mass and recalculated weekly. Given the generally elevated BMI of the sample, use of actual body mass may have resulted in relatively high absolute protein prescriptions for some participants and may limit direct comparison with studies using ideal, adjusted, or lean body mass. Protein sources were self-selected and were not standardised or quantified as animal- versus plant-derived protein. Thus, differences in protein quality, leucine content, digestibility, meal composition, and food substitutions may have contributed to dietary heterogeneity.

Dietary entries recorded in MyFitnessPal were manually transferred to Diet Analysis Plus, and foods not available in the database were represented using manufacturer information or nutritionally similar database items. Mixed dishes required recipe decomposition, and portion sizes were estimated using household measures, package information, or standard serving references. These transcription, substitution, recipe-coding, and portion-estimation procedures introduce additional sources of measurement error. Independent duplicate dietary coding was not performed.

Because outcome data were unavailable for participants who withdrew, the analyses were complete-case analyses and may be vulnerable to attrition-related bias if withdrawal was related to outcomes or participant characteristics. Twenty-five of 108 randomised participants did not complete follow-up testing, corresponding to 23.1% attrition. Findings should therefore be interpreted as randomised-condition comparisons among participants with observed follow-up data and may not reflect the effect of randomised assignment in the full randomised sample. Baseline characteristics for participants who withdrew were unavailable in the final analytic dataset; therefore, completers and noncompleters could not be compared. No outcome values were imputed.

Although the final complete-case sample met the a priori sample-size requirement, the calculation assumed a moderate-to-large training-condition × protein-target interaction (Cohen’s f = 0.35). The study may therefore have had limited sensitivity to detect smaller but potentially relevant marginal protein effects or interactions. Nonsignificant protein findings should be interpreted as an absence of detectable differences under the present conditions rather than as evidence of equivalence, proof of sufficiency, or evidence that protein intake has no influence on adaptation. Selected exploratory findings were also sensitive to the covariance specification in HC3 sensitivity analyses and should not be interpreted as confirmatory.

The CT groups completed the same RT programme as the RT groups followed by additional cycling. Therefore, total exercise duration was greater and exercise-related energy expenditure was likely greater in the CT condition. Exercise duration, total work, internal load, and energy expenditure were not matched between training conditions. Consequently, observed differences cannot isolate the independent biological effect of “training modality” or cycling from differences in overall exercise exposure, session duration, accumulated fatigue, and recovery requirements.

Although the exercise sessions were supervised and the prescribed programmes were described, achieved training exposure was not comprehensively quantified in the reported results. Group-specific attendance, completed sets and repetitions, achieved resistance-training loads, session duration, cycling minutes, cycling workload, heart-rate responses, and deviations from the prescribed progression were not fully available for comparison. It therefore cannot be confirmed that the achieved RT stimulus was equivalent between the RT and CT conditions or that participants consistently completed the prescribed cycling dose.

Aerobic fitness was estimated using the submaximal Ekblom–Bak cycle test rather than measured directly using respiratory-gas analysis during a maximal test. Changes in submaximal heart-rate response, cycling familiarity, pedalling economy, and test-specific adaptation may therefore have influenced the estimated VO₂max values. This concern is particularly relevant because regular cycling was included only in the CT condition, whereas the outcome was also assessed on a cycle ergometer. The findings should therefore be interpreted as changes in cycle-derived estimated VO₂max rather than directly measured maximal oxygen uptake or direct evidence of cardiac or vascular adaptation. The 12-week duration and applied RT progression may have limited the magnitude of hypertrophic adaptation and the ability to detect small incremental protein effects on SMM. Because participants were previously untrained, conservative initial loading and gradual progression were appropriate for safety and technique acquisition. However, achieved loading intensity and proximity to muscular failure were not comprehensively quantified, making it difficult to determine the magnitude of the actual hypertrophic stimulus. Previous training status may also have influenced early responsiveness, but trained and untrained participants were not directly compared. The findings may therefore not generalise to resistance-trained women who have completed the initial phase of adaptation to a novel programme. Whether differences among assigned protein targets would emerge during a longer intervention, following training habituation, or under a more hypertrophy-focused programme remains unknown.

Menopausal status and menopausal hormone therapy use were not assessed using standardised criteria. Consequently, menopausal stage could not be characterised or included in the analyses, and the age bands presented in Table 3 should be interpreted only as descriptive, nonclinical categories. The broad age range of 40–77 years likely encompassed substantial heterogeneity in reproductive aging, physiological function, training responsiveness, and dietary requirements. This heterogeneity, combined with the selected eligibility criteria and the predominantly obesity-range BMI of the sample, limits generalisability to the broader population of middle-aged and older women.

Participants reported no medication use at enrolment, whereas menopausal hormone therapy was not specifically recorded. It is therefore unclear whether the medication-screening question explicitly included hormonal therapies, and unrecorded hormone use cannot be excluded. Sleep disturbances were screened through self-reported diagnosed disorders and/or prescription sleep-medication use; however, subclinical or undiagnosed sleep problems may have been present and could have influenced recovery or adaptation.

No intervention-related adverse events were reported; however, the methods used to actively solicit, classify, and assess the severity and relatedness of adverse events were not fully standardised or described. The absence of reported events should therefore be interpreted cautiously. Finally, the study did not include postintervention follow-up, and the persistence of the observed differences, longer-term adherence to the assigned dietary conditions, and long-term safety or functional outcomes remain unknown.

### 4.7. Practical and mechanistic implications

The primary conventional analyses detected no consistent additional differences among the assigned protein-target conditions for SMM, resistance-test performance, functional performance, or cycle-derived estimated VO₂max. These findings relate to free-living dietary-counselling conditions supported by self-reported intake and should not be interpreted as evidence that objectively verified protein intakes of 0.8, 1.6, and 2.2 g·kg^−1^·d^−1^ are equivalent or that 0.8 g·kg^−1^·d^−1^ is sufficient for all middle-aged and older women. Compared with RT, the CT condition yielded a higher baseline-adjusted cycle-derived estimated VO₂max and lower adjusted BFP, BMI, and body mass, whereas RT yielded higher adjusted leg-press and chest press estimated 1-RM and muscular-endurance performance. These findings represent comparisons between two active intervention packages rather than isolated effects of training modality. The CT condition required additional exercise time and likely greater energy expenditure, whereas the aerobic outcome was assessed using a cycling-specific submaximal test. Practically, RT may be considered when resistance-test strength and muscular endurance are the primary objectives and shorter training sessions are preferred. Adding cycling to the same RT programme may be considered when cycle-derived estimated aerobic fitness and lower adjusted BFP are prioritised and the additional time and recovery demands are acceptable. These recommendations should remain conditional because the interventions were not matched for total exercise exposure and no inactive comparator was included. Mechanistic explanations involving neural adaptation, motor-unit behaviour, muscle-protein synthesis, anabolic signalling, capillary density, mitochondrial enzymes, oxygen extraction, or hormonal pathways remain speculative because these variables were not measured.

## 5. Conclusion

In previously untrained women aged 40–77 years who completed the 12-week intervention, no detectable training-condition × protein-target interaction was observed for SMM or the assessed secondary outcomes in the primary conventional analyses. Small raw increases in SMM were observed across all active conditions, but no baseline-adjusted difference was detected between RT and CT or among the assigned protein targets. These raw changes should not be interpreted as confirmed hypertrophy or causal intervention effects because no nonexercise comparator was included.

Compared with RT, CT yielded a higher baseline-adjusted cycle-derived estimated VO₂max and lower adjusted BFP, BMI, and body mass, whereas RT yielded higher adjusted resistance-test strength and muscular-endurance performance. No detectable training-condition differences were observed for chair-stand or arm-curl performance. Because CT involved additional exercise exposure and the aerobic outcome was estimated using a cycle-based submaximal test, these findings should be interpreted as comparisons between intervention packages rather than isolated modality effects.

No consistent additional differences were detected among the assigned protein-target conditions in the primary analyses. However, these findings were based on self-reported intake under free-living, non-isocaloric conditions and do not establish equivalence among protein intakes, confirm exact dietary adherence, or demonstrate that an RDA-level target was sufficient. Longer controlled studies using objective dietary-adherence measures, direct muscle-imaging methods, directly measured aerobic fitness, and inactive or diet-only comparator conditions are needed to determine whether smaller or longer-term protein-related differences exist.

## Supplementary Material

Supplementary Material Supplementary_Table_S1.docx
