# Supplementary material for: Resistance versus concurrent training with three assigned protein targets in middle-aged and older women: a randomized 2 × 3 factorial trial
Source: J Int Soc Sports Nutr. 2026 Sep 14;23(1):2720742. doi: 10.1080/15502783.2026.2720742 (PMC13577256; doi:10.1080/15502783.2026.2720742)
Supplement: Supplementary file 1 [file RSSN_A_2720742_SM1386.docx]

**Supplementary Table S1. Baseline-adjusted marginal protein-target estimates and exploratory pairwise contrasts.**

**Panel A. Adjusted marginal means by assigned protein target**

| **Tier** | **Outcome** | **0.8 g·kg⁻¹·d⁻¹ adjusted mean (95% CI)** | **1.6 g·kg⁻¹·d⁻¹ adjusted mean (95% CI)** | **2.2 g·kg⁻¹·d⁻¹ adjusted mean (95% CI)** | **Omnibus protein-target p** |
| --- | --- | --- | --- | --- | --- |
| **Primary** | **SMM (kg)** | 25.93 (25.67 to 26.19) | 26.06 (25.81 to 26.31) | 26.05 (25.79 to 26.30) | 0.726 |
| **Key secondary** | **BFP (%)** | 44.91 (44.14 to 45.68) | 45.00 (44.25 to 45.74) | 45.34 (44.56 to 46.11) | 0.715 |
| **Secondary** | **BMI (kg·m⁻²)** | 35.03 (34.48 to 35.57) | 35.27 (34.74 to 35.80) | 35.32 (34.77 to 35.87) | 0.720 |
|  | **Body mass (kg)** | 87.01 (85.65 to 88.38) | 87.65 (86.33 to 88.97) | 87.70 (86.33 to 89.07) | 0.730 |
| **Key secondary** | **30-s chair stand (reps)** | 16.33 (15.98 to 16.68) | 15.97 (15.63 to 16.30) | 15.79 (15.43 to 16.14) | 0.101 |
|  | **Leg press estimated 1-RM (kg)** | 78.07 (76.63 to 79.51) | 79.73 (78.35 to 81.12) | 77.48 (76.04 to 78.91) | 0.069 |
|  | **Cycle-derived estimated VO₂max (mL·kg⁻¹·min⁻¹)** | 25.70 (25.06 to 26.34) | 25.44 (24.82 to 26.05) | 25.19 (24.56 to 25.82) | 0.528 |
| **Secondary** | **30-s arm curl (reps)** | 17.84 (17.51 to 18.17) | 18.03 (17.71 to 18.35) | 17.97 (17.65 to 18.30) | 0.694 |
|  | **Chest press endurance (reps)** | 14.13 (13.76 to 14.51) | 14.54 (14.18 to 14.90) | 14.34 (13.97 to 14.71) | 0.310 |
|  | **Chest press estimated 1-RM (kg)** | 38.62 (37.87 to 39.37) | 38.77 (38.05 to 39.50) | 38.43 (37.68 to 39.18) | 0.807 |
|  | **Leg press endurance (reps)** | 24.46 (24.21 to 24.72) | 24.58 (24.33 to 24.84) | 24.38 (24.11 to 24.64) | 0.536 |

Adjusted marginal means are estimated at the overall mean of the corresponding baseline outcome and averaged equally across the two training conditions. The omnibus p-value is the Type III marginal protein-target effect from the factorial ANCOVA. Confidence intervals are unadjusted pointwise intervals.

**Panel B. Exploratory adjusted pairwise protein-target contrasts**

| **Tier** | **Outcome** | **Protein-target contrast (g·kg⁻¹·d⁻¹)** | **Adjusted mean difference** | **95% CI** | **Raw p** | **Holm-adjusted p** |
| --- | --- | --- | --- | --- | --- | --- |
| **Primary** | **SMM (kg)** | 1.6 − 0.8 | 0.13 | -0.22 to 0.49 | 0.465 | 1.000 |
|  |  | 2.2 − 0.8 | 0.12 | -0.24 to 0.48 | 0.516 | 1.000 |
|  |  | 2.2 − 1.6 | -0.01 | -0.37 to 0.34 | 0.945 | 1.000 |
| **Key secondary** | **BFP (%)** | 1.6 − 0.8 | 0.09 | -0.98 to 1.15 | 0.872 | 1.000 |
|  |  | 2.2 − 0.8 | 0.43 | -0.67 to 1.52 | 0.440 | 1.000 |
|  |  | 2.2 − 1.6 | 0.34 | -0.74 to 1.42 | 0.533 | 1.000 |
| **Secondary** | **BMI (kg·m⁻²)** | 1.6 − 0.8 | 0.24 | -0.52 to 1.00 | 0.526 | 1.000 |
|  |  | 2.2 − 0.8 | 0.29 | -0.48 to 1.07 | 0.451 | 1.000 |
|  |  | 2.2 − 1.6 | 0.05 | -0.71 to 0.81 | 0.896 | 1.000 |
| **Secondary** | **Body mass (kg)** | 1.6 − 0.8 | 0.64 | -1.26 to 2.54 | 0.505 | 1.000 |
|  |  | 2.2 − 0.8 | 0.69 | -1.24 to 2.62 | 0.482 | 1.000 |
|  |  | 2.2 − 1.6 | 0.05 | -1.86 to 1.95 | 0.962 | 1.000 |
| **Key secondary** | **30-s chair stand (reps)** | 1.6 − 0.8 | -0.36 | -0.84 to 0.12 | 0.143 | 0.286 |
|  |  | 2.2 − 0.8 | -0.54 | -1.05 to -0.03 | 0.037 | 0.111 |
|  |  | 2.2 − 1.6 | -0.18 | -0.68 to 0.31 | 0.466 | 0.466 |
| **Key secondary** | **Leg press estimated 1-RM (kg)** | 1.6 − 0.8 | 1.66 | -0.34 to 3.66 | 0.102 | 0.203 |
|  |  | 2.2 − 0.8 | -0.60 | -2.63 to 1.44 | 0.561 | 0.561 |
|  |  | 2.2 − 1.6 | -2.26 | -4.25 to -0.27 | 0.027 | 0.081 |
| **Key secondary** | **Cycle-derived estimated VO₂max (mL·kg⁻¹·min⁻¹)** | 1.6 − 0.8 | -0.26 | -1.15 to 0.63 | 0.558 | 1.000 |
|  |  | 2.2 − 0.8 | -0.51 | -1.41 to 0.39 | 0.260 | 0.779 |
|  |  | 2.2 − 1.6 | -0.25 | -1.13 to 0.63 | 0.577 | 1.000 |
| **Secondary** | **30-s arm curl (reps)** | 1.6 − 0.8 | 0.19 | -0.26 to 0.65 | 0.405 | 1.000 |
|  |  | 2.2 − 0.8 | 0.14 | -0.33 to 0.60 | 0.563 | 1.000 |
|  |  | 2.2 − 1.6 | -0.06 | -0.51 to 0.40 | 0.806 | 1.000 |
| **Secondary** | **Chest press endurance (reps)** | 1.6 − 0.8 | 0.41 | -0.12 to 0.93 | 0.128 | 0.383 |
|  |  | 2.2 − 0.8 | 0.21 | -0.32 to 0.74 | 0.440 | 0.879 |
|  |  | 2.2 − 1.6 | -0.20 | -0.71 to 0.31 | 0.440 | 0.879 |
| **Secondary** | **Chest press estimated 1-RM (kg)** | 1.6 − 0.8 | 0.15 | -0.90 to 1.20 | 0.777 | 1.000 |
|  |  | 2.2 − 0.8 | -0.19 | -1.26 to 0.87 | 0.716 | 1.000 |
|  |  | 2.2 − 1.6 | -0.34 | -1.39 to 0.70 | 0.514 | 1.000 |
| **Secondary** | **Leg press endurance (reps)** | 1.6 − 0.8 | 0.12 | -0.24 to 0.48 | 0.508 | 1.000 |
|  |  | 2.2 − 0.8 | -0.09 | -0.46 to 0.28 | 0.627 | 1.000 |
|  |  | 2.2 − 1.6 | -0.21 | -0.58 to 0.16 | 0.268 | 0.804 |

Contrasts are collapsed across training condition. Positive values favor the higher listed protein target. These pairwise analyses are exploratory because no training-condition × protein-target interaction was statistically significant. Holm adjustment was applied within each outcome across its three pairwise protein-target contrasts; the 95% confidence intervals remain unadjusted pointwise intervals. Nominal raw p-values below 0.05 for the 2.2–0.8 chair-stand contrast and the 2.2–1.6 Leg press estimated 1-RM contrast did not remain below 0.05 after Holm adjustment.

**Supplementary Table S2. Model diagnostics and sensitivity analyses.**

**Panel A. Conventional ANCOVA diagnostics**

| **Outcome** | **Residual Shapiro–Wilk p** | **Levene p** | **Baseline × randomized-group p** | **Cook’s D > 4/N, n** |
| --- | --- | --- | --- | --- |
| SMM | <0.001 | 0.396 | 0.409 | 4 |
| BFP | 0.002 | 0.381 | 0.053 | 6 |
| BMI | 0.712 | 0.177 | 0.330 | 4 |
| Body mass | 0.678 | 0.121 | 0.512 | 3 |
| 30-s chair stand | <0.001 | 0.002 | 0.890 | 4 |
| Leg press estimated 1-RM | 0.432 | 0.036 | 0.222 | 4 |
| Cycle-derived estimated VO₂max | 0.746 | 0.022 | <0.001 | 7 |
| 30-s arm curl | 0.566 | 0.237 | 0.013 | 5 |
| Chest press endurance | 0.811 | 0.467 | 0.123 | 4 |
| Chest press estimated 1-RM | 0.376 | 0.519 | 0.208 | 6 |
| Leg press endurance | 0.604 | 0.192 | 0.347 | 5 |

Shapiro–Wilk tests were applied to model residuals. Levene’s test used the mean-centered version across the six randomized groups. The baseline × randomized-group test evaluates homogeneity of regression slopes. Cook’s D > 4/N is reported descriptively as a screening criterion; it does not by itself establish that an observation should be excluded. Diagnostic findings motivated the sensitivity analyses below.

**Panel B. Conventional and HC3 heteroscedasticity-consistent omnibus p-values**

| **Outcome** | **Interaction conventional p** | **Interaction HC3 p** | **Training condition conventional p** | **Training condition HC3 p** | **Protein target conventional p** | **Protein target HC3 p** |
| --- | --- | --- | --- | --- | --- | --- |
| SMM | 0.856 | 0.879 | 0.273 | 0.294 | 0.726 | 0.699 |
| BFP | 0.998 | 0.998 | <0.001 | <0.001 | 0.715 | 0.741 |
| BMI | 0.990 | 0.991 | <0.001 | <0.001 | 0.720 | 0.737 |
| Body mass | 0.981 | 0.984 | <0.001 | <0.001 | 0.730 | 0.732 |
| 30-s chair stand | 0.488 | 0.474 | 0.178 | 0.208 | 0.101 | 0.048 |
| Leg press estimated 1-RM | 0.084 | 0.052 | <0.001 | <0.001 | 0.069 | 0.040 |
| Cycle-derived estimated VO₂max | 0.995 | 0.996 | <0.001 | <0.001 | 0.528 | 0.609 |
| 30-s arm curl | 0.051 | 0.049 | 0.270 | 0.280 | 0.694 | 0.692 |
| Chest press endurance | 0.331 | 0.364 | <0.001 | <0.001 | 0.310 | 0.396 |
| Chest press estimated 1-RM | 0.646 | 0.693 | <0.001 | <0.001 | 0.807 | 0.815 |
| Leg press endurance | 0.051 | 0.063 | <0.001 | <0.001 | 0.536 | 0.540 |

HC3 analyses used heteroscedasticity-consistent covariance estimates while retaining the same factorial ANCOVA mean structure. The primary SMM conclusion and all principal training-condition findings were unchanged. Selected exploratory results were sensitive to covariance specification: the HC3 protein-target p-values were 0.048 for chair-stand performance and 0.040 for Leg press estimated 1-RM, and the HC3 training-condition × protein-target interaction p-value was 0.049 for 30-s arm-curl performance.

**Panel C. Group-specific baseline-slope sensitivity analyses**

| **Outcome** | **Adjusted difference (CT − RT)** | **95% CI** | **p** |
| --- | --- | --- | --- |
| Cycle-derived estimated VO₂max (mL·kg⁻¹·min⁻¹) | 4.62 | 3.98 to 5.26 | <0.001 |
| 30-s arm curl (reps) | −0.22 | −0.57 to 0.14 | 0.232 |

Group-specific baseline-slope models were examined because the homogeneity-of-regression-slopes test was significant for cycle-derived estimated VO₂max and 30-s arm-curl performance. Adjusted contrasts were evaluated at the overall mean of the corresponding baseline outcome and averaged equally across the three protein targets. These sensitivity models did not materially change the training-condition conclusions.

**Panel D. Exploratory HC3 pairwise protein-target sensitivity contrasts**

| **Outcome** | **Protein-target contrast (g·kg⁻¹·d⁻¹)** | **Adjusted mean difference** | **HC3 95% CI** | **Raw p** | **Holm-adjusted p** | **N** |
| --- | --- | --- | --- | --- | --- | --- |
| 30-s chair stand (reps) | 2.2 − 0.8 | −0.54 | −1.02 to −0.06 | 0.028 | 0.085 | 83 |
| Leg press estimated 1-RM (kg) | 2.2 − 1.6 | −2.26 | −4.07 to −0.45 | 0.015 | 0.046 | 83 |

HC3 pairwise contrasts were collapsed across training condition and adjusted within each outcome across the three protein-target comparisons using Holm’s method. The chair-stand contrast did not remain below 0.05 after adjustment, whereas the Leg press estimated 1-RM contrast was below 0.05 in this exploratory sensitivity analysis. These findings were model-sensitive and were not treate
